# A machine learning-based prognostic model integrating mRNA stemness index, hypoxia, and glycolysis‑related biomarkers for colorectal cancer

**DOI:** 10.1515/med-2025-1247

**Published:** 2025-09-15

**Authors:** Dan Liu, MingLong Zhang, Ying Nie, XingNan Li, WanQuan Liu, LiLing Yue, XianDong Meng, PengHui Li, LuLu Wang, QingBu Mei

**Affiliations:** Department of Medical Genetics, School of Basic Medicine, Qiqihar Medical University, Heilongjiang, 161006, China; Department of Foreign Language, Qiqihar Medical University, Qiqihar, Heilongjiang, 161006, China; Department of Dermatology, Weihai District of the 970th Hospital of the People’s Liberation Army Joint Logistic Support Force, Weihai, Shandong, 264200, China; Biotechnology Experimental Teaching Center, School of Basic Medicine, Qiqihar Medical University, Qiqihar, Heilongjiang, 161006, China; Department of Anatomy, School of Basic Medicine, Qiqihar Medical University, Qiqihar, Heilongjiang, 161006, China

**Keywords:** colorectal cancer, mRNAsi, stemness, hypoxia, glycolysis, prognostic model

## Abstract

**Background:**

Cancer stemness, hypoxia, and glycolysis collectively influence colorectal cancer (CRC) progression. However, the intricate relationships among these factors remain incompletely understood.

**Methods:**

This study (1) explored hypoxia and glycolysis-related genes (HGRGs) in CRC by mRNA stemness index (mRNAsi), analyzed the gene expression profiles from Gene Expression Omnibus and The Cancer Genome Atlas (TCGA) databases, (2) established a Cox-prognostic model based on single-sample gene set enrichment analysis, differentially expressed gene analysis, weighted gene co-expression network analysis, and Least Absolute Shrinkage and Selection Operator (LASSO) and Cox regression analyses, and (3) assessed the predictive accuracy of the model. Decision curve analysis (DCA) was employed to determine the clinical utility of the model.

**Results:**

Ten HGRGs were selected based on mRNAsi to create the LASSO model. High-risk CRC patients in the TCGA dataset displayed unfavorable clinical outcomes and responses to immunotherapy. Consensus cluster analysis revealed two distinct colon adenocarcinoma/rectal adenocarcinoma clusters, with patients in cluster 2 having a worse prognosis compared to patients in cluster 1. A five-gene prognostic nomogram was developed through univariate and multivariate Cox regression analyses, with DCA confirming its accuracy.

**Conclusions:**

This innovative prognostic model, incorporating *ALDOB*, *AQP1*, *IL1A*, *PHGDH*, and *PTGIS*, is highly accurate in predicting patient survival.

## 1 Introduction

Colorectal cancer (CRC) is the third most dominant global malignancy, profoundly impacting the gastrointestinal tract [1]. Despite multiple treatment modalities, including radiotherapy, targeted therapy, chemotherapy, surgery, immunotherapy, and traditional Chinese medicine, which have shown promise in extending patient survival, CRC continues to stand as the second most prominent contributor to cancer-related mortality, primarily attributing to early metastasis, late-stage diagnosis, and rapid progression [1,2,3,4]. Therefore, it is urgent to identify novel therapeutic targets and early diagnostic markers for CRC. CRC is a heterogeneous disease influenced by complex genomic variation and abnormal biological microenvironments [5]. Single-gene/factor prediction models often have limitations, whereas multigene-based models show excellent predictive power across a wide range of cancers [6,7,8]. Therefore, there is an urgent need to identify reliable genetic profiles for early CRC diagnosis, prognostic assessment, and targeted therapy.

Cancer stem cells (CSCs) represent a specialized and rare cell population present within tumor cell communities. They are self-renewing and play a key role in tumour formation, metastasis, recurrence, and resistance to therapeutic agents, with important implications for tumor progression and prognosis [9,10]. In CRC, CSCs are identified and defined by specific markers, including CD133, CD24, CD166, Lgr5, EpCAM, CD44, CD29, ALDH1, and β-catenin [11,12]. Recently, sequencing data analysis has emerged as a key approach to cancer prognosis assessment, which provides a new analytical perspective by characterizing cancer stemness using stemness-associated genes and the mRNA stemness index (mRNAsi) derived from machine learning algorithms [13]. Studies have explored the application of stemness scores in predicting prognosis, assessing immunotherapy response, and evaluating clinical outcomes in cancers, including glioblastoma, lung cancer, cutaneous melanoma, and CRC [14,15,16,17].

Hypoxia is a common feature of most solid tumors, resulting from increased oxygen consumption and vascular disturbances, and is frequently associated with a poor prognosis [18,19]. Hypoxia has profound effects on intracellular and extracellular metabolic processes, triggering the activation of hypoxia-inducible factors. These factors in turn stimulate the transcription of important genes involved in various processes such as angiogenesis, pH regulation, glucose metabolism, tumor invasion, and metastasis [20,21]. Moreover, recent research has shown that a hypoxic tumor microenvironment can influence the efficacy of chemotherapy in CRC patients [22,23]. In CRC cells, a modified energy metabolism, particularly abnormal activation of the glycolytic pathway, has been observed [24,25]. In the presence of sufficient oxygen, cancer cells rely mainly on glycolysis to produce energy [26]. In cancer cells, glycolysis accounts for the production of 50–60% of total adenosine triphosphate [27]. Aerobic glycolysis creates a specific microenvironment that favours the unrestricted growth and invasion of cancer cells [28]. Currently, investigations have developed hypoxia and glycolysis (HG)-related prognostic signatures for CRC patients [29,30], and hypoxia and glycolysis-related genes (HGRGs) are anticipated to serve as valuable prognostic biomarkers for CRC.

The main focus of the study was to identify HGRGs by analyzing tumor stemness in CRC patients, to study their pattern in normal and tumour tissues, and to use them as potential prognostic markers. We identified 30 HGRGs related to mRNAsi and validated their expression levels using The Cancer Genome Atlas (TCGA) and Gene Expression Omnibus (GEO) datasets. Further insights into their functional implications were gained through Gene Ontology (GO) and Kyoto Encyclopedia of Genes and Genomes (KEGG) pathway analyses. A prognostic model comprising ten genes was developed using the Least Absolute Shrinkage and Selection Operator (LASSO) model. Consensus clustering of these genes identified two colon adenocarcinoma/rectal adenocarcinoma (COADREAD) subtypes and compared their survival, tumor mutational burden (TMB), and microsatellite instability (MSI) in the Tumour Immune Dysfunction and Rejection (TIDE) database. Finally, a prognostic nomogram incorporating five genes was introduced through LASSO–Cox regression analysis, and its predictive performance was verified using decision curve analysis (DCA). The DCA curve takes the sensitivity and specificity of the predictive model as the horizontal axis and the benefit as the vertical axis. If the decision curve of the predictive model is above the decision curve of the reference strategy, the clinical utility of the predictive model is higher; conversely, the clinical utility is lower. In conclusion, this study enriches our understanding of the pathogenesis of CRC and paves the way for personalized and tailored patient care.

## 2 Materials and methods

### 2.1 Data manipulation

The gene expression matrix data for COADREAD and relevant clinical details were retrieved from TCGA (https://portal.gdc.cancer.gov/) and UCSC Xena (http://genome.ucsc.edu), respectively [31,32]. The raw read counts were converted to Fragments Per Kilobase per Million using the R package limma [33]. Samples lacking key clinical data were eliminated, yielding in a final dataset comprising 644 cancer samples and 51 normal samples. Validation datasets for COADREAD were obtained from GSE14333 [34], GSE74602 [35], GSE87211 [36], and GSE161158 [37] from the GEO database [38] using the GEOquery package [39].

Hypoxia-related genes with a relevance score of >1 as the screening criterion were collected from GeneCards, a database containing extensive information about human genomes [40], using the term “hypoxia” as the primary search keyword, resulting in 2041 genes. We also collected 3147 hypoxia-related genes from 65 reference gene sets in MSigDB [41] using “hypoxia” as the keyword. Additionally, we searched the AmiGO2 website (http://amigo.geneontology.org/amigo) [42] using “hypoxia” as the keyword and obtained 298 hypoxia-related genes. By considering the common genes identified across these three sources, we collected 130 hypoxia-related genes. Similarly, we searched the GeneCards database, using the term “glycolysis” to compile a list of glycolysis-related genes and retrieved 851 genes with a relevance score of >1 as the screening criterion. Additionally, we found 753 glycolysis-related genes from 21 reference gene sets in MSigDB using “glycolysis” as the search term. By considering the common genes across these two sources, we collected 158 glycolysis-related genes. Ultimately, we obtained a subset of 273 HGRGs. Additional details can be found in Table S1.

In addition to gene expression data, we retrieved somatic mutation data, including single-nucleotide polymorphism (SNP), for COADREAD from TCGA and processed the data with the R package maftools [43]. These genomic characterizations were designed to explore the potential association between the pattern of genetic variation in HGRG-related genes and their expression levels and prognosis, and to assess the impact of genomic instability on immunotherapy response. Specifically, (1) analysis of somatic mutation types and frequencies by maftools can reveal the relationship between the mutational load of HGRG-related genes and patient prognosis; (2) copy number variation (CNV) analysis (GISTIC 2.0) can help to identify the genomic amplification/deletion events that drive the expression of HGRG; and (3) analysis of the MSI/TMB data in combination with the TIDE scores can assess the genomic instability’s role in the regulation of immune escape. Information regarding TMB and MSI in the COADREAD dataset was acquired from cBioPortal (https://www.cbioportal.org/) [44].

### 2.2 Dataset normalization and merging

We normalized COADREAD datasets from both TCGA and GEO using the R package limma. We then used the R package sva to remove the batch effects found in the COADREAD datasets (GSE14333, GSE74602, GSE87211, and GSE161158) from GEO and created a merged COADREAD dataset. The differences between the GEO datasets pre- and post-processing were illustrated in the distribution box and principal component analysis (PCA) plots.

### 2.3 Stemness index calculation

The mRNAsi for each sample was calculated using the stemness training dataset by single-sample gene set enrichment analysis (ssGSEA). The mRNAsi score for COADREAD patients was computed in accordance with the expression profile of the TCGA-COADREAD dataset with the R package GSVA [45]. The resulting score enabled the categorization of cancer samples into separate groups and facilitated correlation analysis.

### 2.4 Differentially expressed gene (DEG) analysis

The individuals afflicted with COADREAD from TCGA were stratified into two mRNAsi cohorts with a median mRNAsi score as the threshold. Subsequently, DEGs between the two groups were uncovered based on *P* < 0.05 and |log_2_ fold change (FC)| > 0. Genes with Log2 FC > 0 or < 0 are considered up-regulated and down-regulated genes, respectively. Finally, we obtained mRNAsi-related HGRGs for subsequent analysis by taking the intersection of DEGs and HGRGs.

### 2.5 Gene set enrichment analysis (GSEA)

We conducted GSEA [46] using the R package clusterProfiler [47] to examine DEGs distribution pattern and determine their impacts on phenotypes based on the c2.cp.v7.2 symbol reference in the MSigDB. The GSEA parameters were configured as: seed value of 2020, calculation number of 100,000, minimum of five genes per set, and a maximum of 500 genes. *P*-values were corrected using the Benjamini–Hochberg method to assess the statistical significance of enrichment. Gene sets with *P* < 0.05 and false discovery rate (FDR) (*q* value) <0.25 were considered significant enrichment.

### 2.6 HGRG score calculation

The abundance of individual genes within the dataset was assessed using the ssGSEA method. The HGRG score for each sample was computed according to the TCGA-COADREAD levels and mRNAsi-related HGRGs using the ssGSEA algorithm from the R package GSVA and used for sample grouping and correlation analysis. COADREAD patients were divided into two groups, using the median HGRGs score as the cut-off value.

### 2.7 Weighted gene co-expression network analysis (WGCNA)

WGCNA [48] was employed to delineate gene correlation patterns across multiple samples. Highly coordinated gene sets and potential biomarkers or therapeutic targets were identified by examining their connectivity and correlations with phenotypes using the R package WGCNA [49]. Correlation coefficients were first calculated for any two genes, and then weighted correlation coefficients were applied to ensure a scale-free distribution of gene connections within the network. Subsequently, a hierarchical clustering tree between genes was built based on the correlation between genes. Different gene modules were used to describe different branches of the tree based on unique colors, and the importance of each module was assessed. The top 25% of genes with the most pronounced differences in gene expression in the ARDS dataset were entered with a minimum number of 50 genes, a soft power threshold of 10, no module merging (shear height of 0), and a minimum distance of 0.2. Following this, the correlation between the HGRGs scores and various modules was computed. Simultaneously, genes in each module were considered signature genes. Prominent modules exhibiting strong correlation coefficients were selected, and their intersection with mRNAsi-related HGRGs was used as hypoxia and glycolysis-related module DEGs (HGRMDEGs) for further analysis.

### 2.8 GO and KEGG annotation analyses

The expression levels of HGRMDEGs were validated using a validation set, and genes with inconsistent validation results were excluded. The HGRMDEGs were annotated by GO [50] and KEGG [51] analyses using the R package clusterProfiler based on *P* < 0.05 and an FDR value (*q* value) of <0.25 and the Benjamini–Hochberg method.

### 2.9 LASSO model

LASSO regression was used to construct prognostic models to address the risk of overfitting in high-dimensional genetic data. Tenfold cross-validation (seed = 2,020) assessed model stability through repeated sampling, and a penalty term of *λ* = 0.01 prevented model complexity while preserving key genes. A median risk score grouping strategy maximized survival differences between the two groups to ensure clinical translational value.

### 2.10 Immunotherapy analysis

Gene expression profile was analyzed based on the TIDE database [52] to determine the likelihood of tumor immune evasion. Elevated TIDE scores indicate an increased likelihood of immunosurveillance evasion and reduced success of immunotherapy. Sample matrices from the TCGA-COADREAD dataset were uploaded to obtain TIDE scores for each COADREAD patient and analyzed for their association with HGRGs.

### 2.11 Protein–protein interaction (PPI) analysis

PPI network of HGRMDEGs between known and predicted proteins was predicted by searching the STRING [53] with a minimum interaction score of 0.4 to ensure moderate confidence. Local regions with close connections were identified by visualizing the PPI network using Cytoscape software (version 3.9.1) [54]. The GeneMANIA website [55] was utilized to predict genes functionally similar to the target genes and to construct an interaction network of HGRMDEGs from the LASSO model. miRNA interactions with HGRMDEGs were predicted using the ENCORI database [56]. Subsequently, miRNAs with supported database counts greater than three were filtered out, and the mRNA–miRNA interaction was analyzed using Cytoscape software.

### 2.12 Consensus clustering

Consensus clustering [57], a method based on resampling, was utilized to establish consensus and evaluate the stability of clusters identified using a clustering algorithm. Using the R package ConsensusClusterPlus, different COADREAD disease subtypes were identified based on HGRMDEG [58]. In this procedure, the cluster counts ranged from 2 to 8, and the entire sample set was repeated 1,000 times at a sampling rate of 80%. clusterAlg parameter was set to “km” (k-means algorithm), and the distance parameter was set to “euclidean.”

### 2.13 Drug sensitivity analysis

To assess the potential sensitivity of CRC patients in the TCGA cohort to commonly used anticancer drugs, a computational pharmacology approach was used. The Genomics of Drug Sensitivity in Cancer (GDSC) database v2.0 (https://www.cancerrxgene.org/) was first used as a training set, which contains drug dose-response data (IC50 values) and gene expression profiles of human cancer cell lines. Then, a drug sensitivity prediction model was constructed by a ridge regression algorithm based on gene expression data (RNA-seq or microarray) from the GDSC training set with IC50 values using the pRophetic R software package. For each drug, pRophetic performed feature selection (screening the most relevant genes based on the correlation between gene expression and IC50), model training (constructing a ridge regression model using the screened gene features and cell line IC50 values), and cross-validation (evaluating the model performance with Pearson’s correlation coefficient and RMSE using LOOCV or k-fold cross-validation on the GDSC training set and recording the average metrics). Subsequently, the trained model was applied to the gene expression profiles of TCGA samples (which should be consistent with the detection platform and normalization method of the GDSC training set), and the predicted IC50 values of each sample for different drugs were output by pRophetic (the lower the IC50, the higher the sensitivity). Finally, the TCGA samples were grouped according to HGRG scores (or variables such as risk group), and the Wilcoxon rank-sum test was used to compare the differences in predicted IC50 between the groups, screen for significant drugs (*P* < 0.05), and visualize the IC50 distribution.

### 2.14 Immune activity analysis

Estimating Stromal and Immune Cells in Malignant Tissue Using Expression Data (ESTIMATE) uses gene expression profiles of tumor samples to detect the relative proportions of tumor cells and infiltrating normal cells. It also quantifies immune activity to derive an immune score. To gain insight into the prognostic impact of genes associated with stromal and immune cells, we analyzed the immune response of tumors using expression profiles from the TCGA-COADREAD dataset and using the R package ESTIMATE [59]. In addition, we quantified the immune activity in the cancer samples using gene expression profiling to derive an immune score for each cancer sample. Subsequently, we assessed the association between the immune infiltration profile of COADREAD patients and the HGRG score.

### 2.15 Immune infiltration analysis

CIBERSORTx is an online tool for analyzing immune cells utilizing a deconvolution algorithm to examine the relative fraction of immune cells in cancer tissues. We provided the grouped gene expression data to the CIBERSORTx website, which was then combined with a signature gene matrix called LM22 to produce immune cell infiltration matrix data. We further analyzed the transcriptome data and accurately quantified the immune and stromal cells in the tissue using the R package MCPcounter [60]. In addition, we calculated an enrichment score for each sample using the ssGSEA algorithm to reflect the overall infiltration level of 28 types of immune cells.

### 2.16 Cox model

To examine the clinical prognostic value of HGRMDEGs obtained from the LASSO prognostic model in COADREAD, we performed univariate Cox regressions using the TCGA-COADREAD dataset to examine the expression pattern in relation to clinical variables. Multivariate Cox regression was examined for variables with *P* < 0.1, and the optimal set of variables was determined by stepwise regression. Based on the Cox model, we generated forest plots and nomograms to estimate 1-, 3-, and 5-year survival for COADREAD patients and built calibration curves using the R package rms to determine their accuracy. We built DCA plots using the R package ggDCA [61] to assess the performance of the nomogram model in predicting survival in COADREAD patients.

### 2.17 Statistical analysis

Drug sensitivity and all other data were assessed using R software. Continuous variables were shown as the median ± quartile interval, and the differences between the two groups were examined using the Wilcoxon rank sum test. The statistical significance in normally distributed variables and categorical variables between two groups was analyzed using the Chi-square or Fisher’s exact tests. Differences among different groups were examined with the Kruskal–Wallis test. LASSO regression analysis was conducted with the R package glmnet [62]. Receiver operating characteristic curve (ROC) and the time-dependent ROC [63] were established with the R package pROC and timeROC, respectively. Survival curves were analyzed with the R package survival and visualized with the survminer package. Significant variations in survival time between the two groups were evaluated with the log-rank test. Spearman’s rank correlation analysis was used to evaluate correlation coefficients between two numerators or scores, unless specified otherwise. All *P* values were two-tailed, and *P* < 0.05 was determined as statistical significance.

## 3 Results

### 3.1 Flow chart

**Figure 1:**
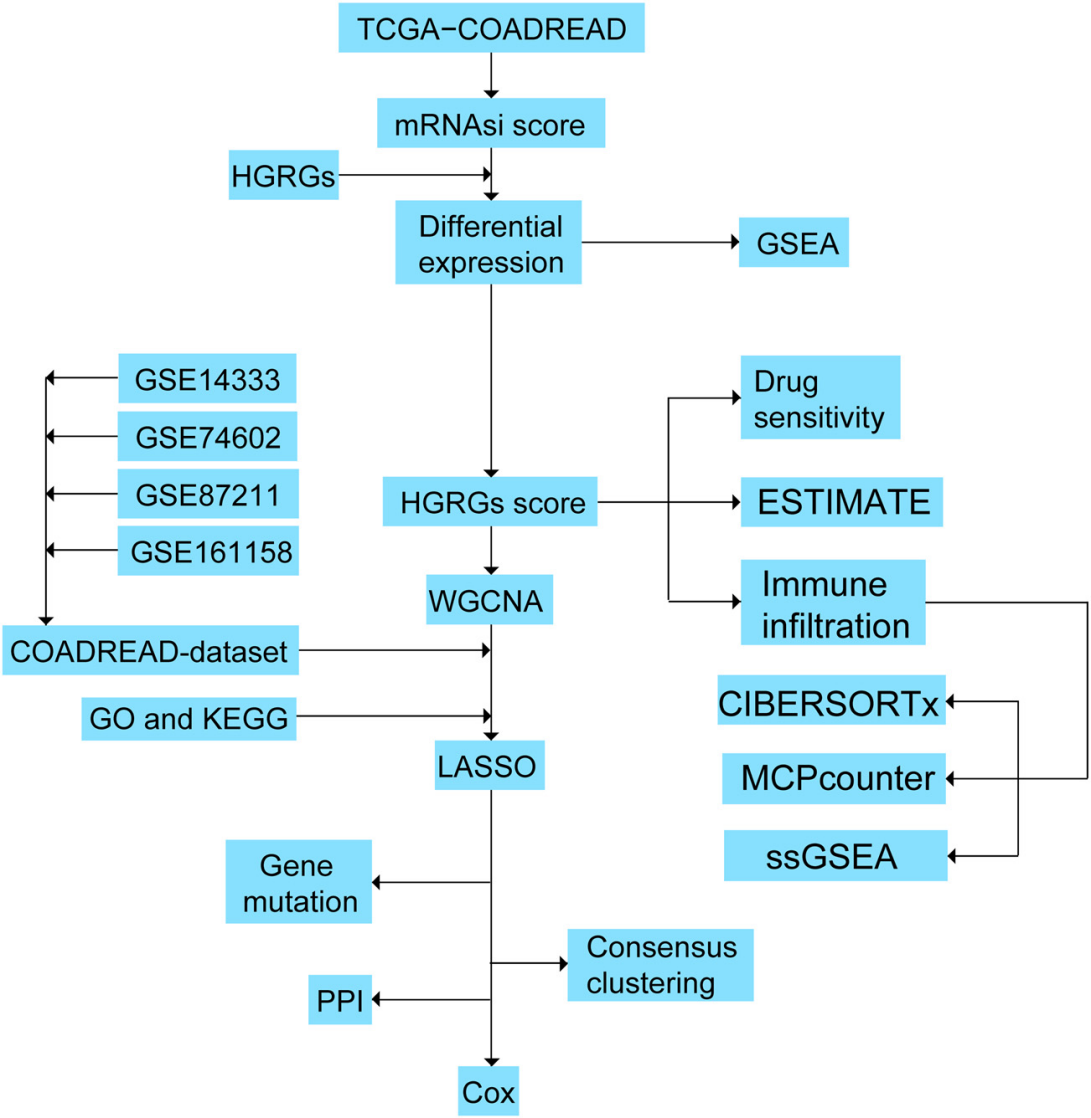
Study flow chart.

The workflow is shown in Figure 1.

### 3.2 Stemness-related gene screen reveals 198 HGRGs

We first created a merged COADREAD dataset by eliminating the batch effect in the GEO-COADREAD datasets (GSE14333, GSE74602, GSE87211, and GSE161158). We then used distribution box plots and PCA plots to compare the datasets before and after correction (Figure S1a–d). These visual illustrations clearly show that the samples in the COADREAD dataset successfully eliminate the batch effect.

**Figure 2:**
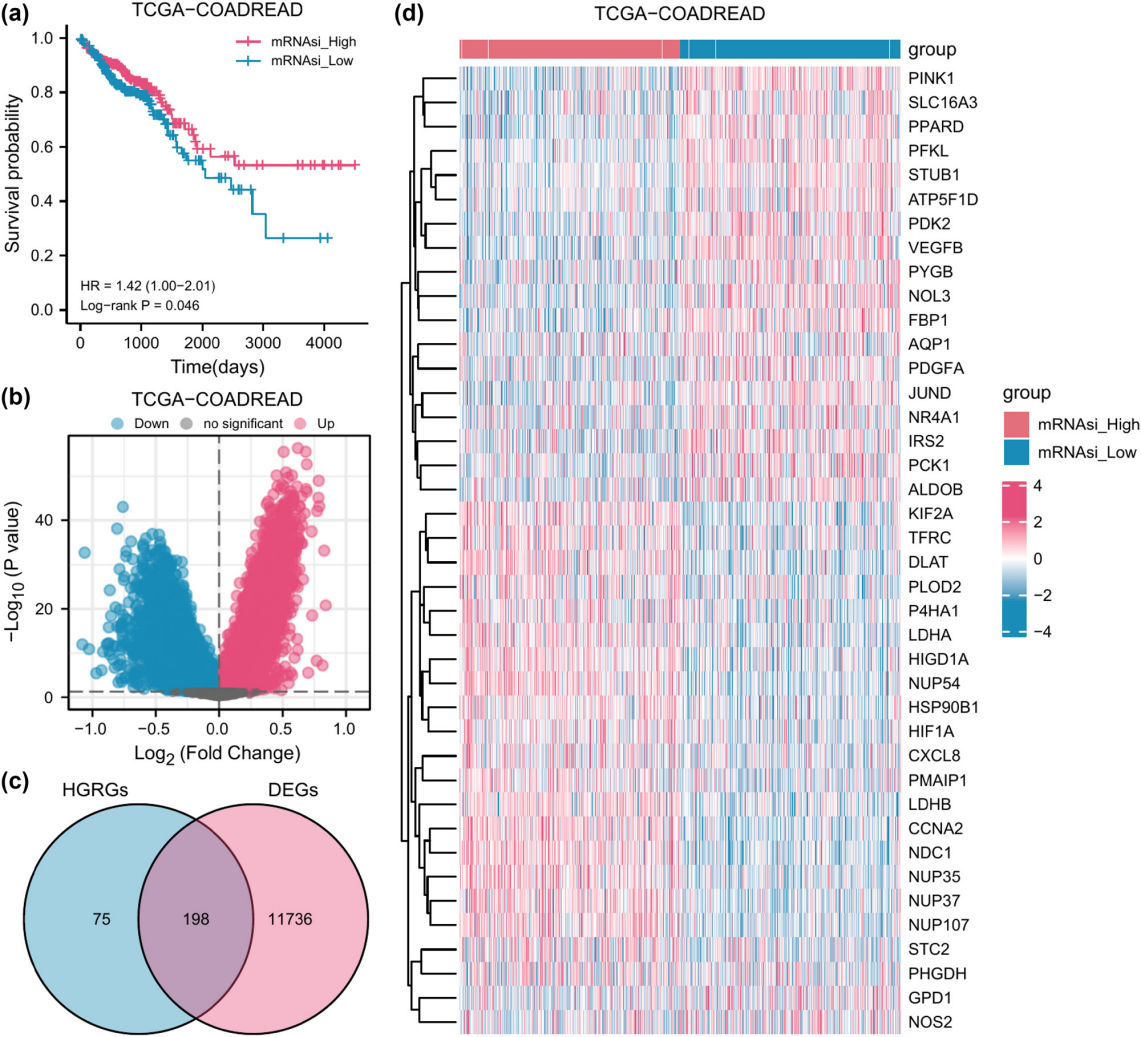
Screening of mRNAsi-related HGRGs in COADREAD patients. (a) The Kaplan–Meier survival curve of the two mRNAsi groups. (b) Volcano plot of DEGs between the two mRNAsi groups. (c) The overlaps of HGRGs and DEGs. (d) The heat map of the top 40 DEGs.

We calculated mRNAsi values for all patients in the TCGA-COADREAD dataset using the ssGSEA algorithm to assess their stemness levels. Subsequently, we divided these patients into two groups based on the median mRNAsi value. We plotted Kaplan–Meier curves (Figure 2a) and found a statistically significant difference in the prognosis of these two groups of patients, with the low mRNAsi group having a worse prognosis. Among the 19,572 genes in the TCGA-COADREAD dataset, we identified 11,934 DEGs, of which 6,388 genes were overexpressed and 5,546 genes were underexpressed in the high mRNAsi group (Figure 2b). We intersected DEGs associated with mRNAsi with HGRGs and obtained 198 HGRGs associated with mRNAsi, as shown in the Venn diagram (Figure 2c). These genes are shown in Table S2, and the first 40 genes are shown in the heatmap.

### 3.3 Hypoxia, glycolysis, and oncogenic pathways drive CRC stemness

**Figure 3:**
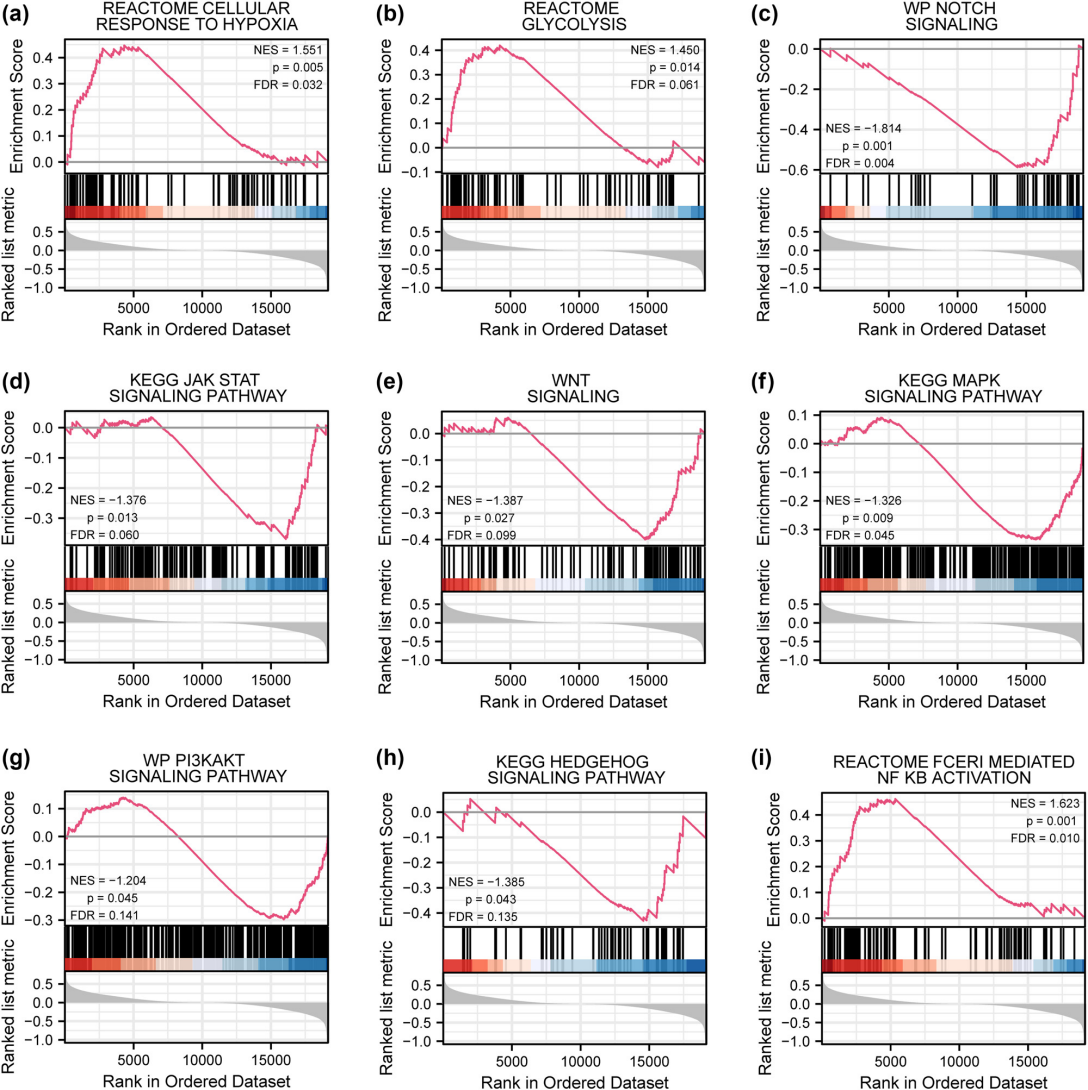
Hypoxia, glycolysis, and oncogenic pathways drive CRC stemness. Genes in the TCGA-COADREAD dataset were significantly enriched in the cellular response to hypoxia (a), glycolysis (b), Notch (c), JAK-STAT (d), Wnt (e), MAPK (f), PI3K-AKT (g), Hedgehog (h), and FcεRI-mediated NF-кB (i) signaling pathways.

We identified biological functions and pathways associated with these DEGs by GSEA. Several significantly enriched pathways can be seen in the pathway map (Figure 3a–i) and are listed in Table S3. Among them, cellular response to hypoxia (Figure 3a) and glycolytic pathway (Figure 3b) were associated with HG phenotypes, whereas Notch (Figure 3c), JAK-STAT (Figure 3d), Wnt (Figure 3e), MAPK (Figure 3f), PI3K-AKT (Figure 3g), Hedgehog (Figure 3h), and FcεRI-mediated NF-кB activation pathway (Figure 3i) were associated with hotspot molecules.

### 3.4 WGCNA analysis and HGRG score cross-validation reveal 50 core stemness-regulated genes in CRC

**Figure 4:**
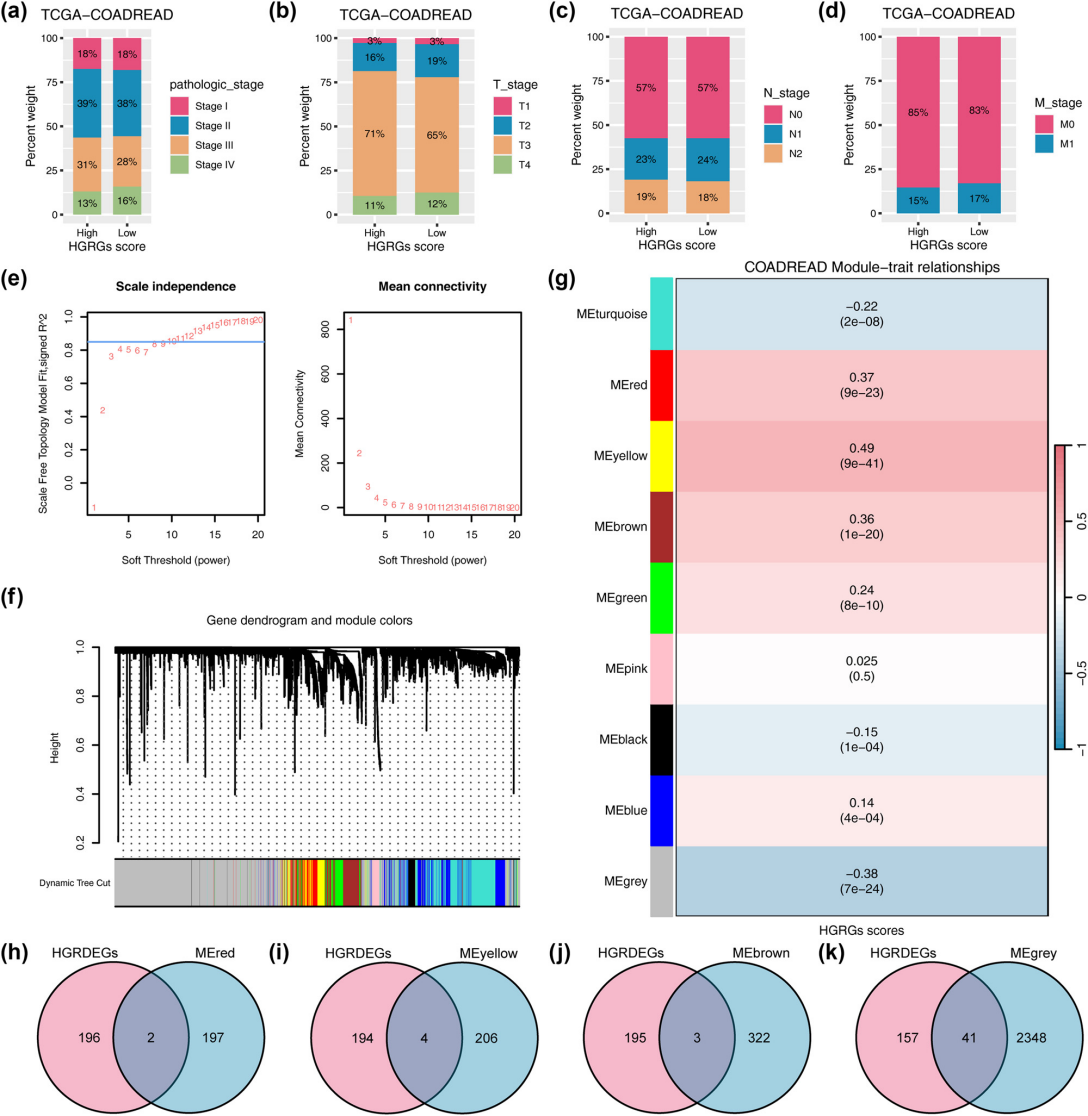
WGCNA analysis and HGRG score cross-validation reveal 50 core stemness-regulated genes in CRC. The proportions of the clinical-pathological (a), as well as clinical T (b), M (c), and N (d) stages in the two HGRG score groups. (e) The scale-free network of optimal soft thresholding power screening in the TCGA-COADREAD dataset. (f) Gene dendrogram and module colors. (g) The relationship between module eigengenes and HGRGs scores in the TCGA-COADREAD dataset. The Venn diagrams showing the overlaps between HGRMDEGs and the genes in the MEred module (h), MEyellow module (i), MEbrown module (j), and MEgrey module (k) of TCGA-COADREAD dataset.

Based on the levels of the 198 mRNAsi-associated HGRGs (Table S2), we calculated HGRG scores for all cancer samples in the TCGA-COADREAD dataset using the ssGSEA algorithm. We created a stacked bar chart to visualize the distribution of all clinical stages in the high and low HGRGs subgroups (Figure 4a–d). Our analyses showed no significant differences between the two groups, except for some changes observed in the T stage relative to the N and M stages. We further performed WGCNA analysis on all genes in the dataset and identified the top 25% of genes with the most significant differences between patients by setting the screening threshold criterion to 0.85 (Figure 4e). These genes were further clustered into nine modules by setting the optimal soft threshold to 10 (Figure 4f and g) and the branch merge cutoff height to 0, i.e., no modules were merged (Figure 4f). Subsequently, we analyzed the correlation between their expression patterns in these modules and the HGRGs scores of COADREAD patients (Figure 4g). Four modules with robust correlations (*P* < 0.05, |*r*| ≥ 0.30), namely MEred (*r* = 0.37, *P* = 9 × 10^−23^), MEyellow (*r* = 0.49, *P* = 9 × 10^−41^), MEbrown (*r* = 0.36, *P* = 1 × 10^−20^), and MEgrey (*r* = −0.38, *P* = 7 × 10^−24^), were selected and further analyzed. We then intersected the 198 mRNAsi-related HGRGs with the genes in the MEred module (Figure 4h), MEyellow module (Figure 4i), MEbrown module (Figure 4j), and MEgrey module (Figure 4k) of the TCGA-COADREAD dataset. We identified 50 HGRMDEGs and presented them in Venn diagrams (Table S4).

### 3.5 Chromosomal localization and PPI network analysis reveal functional clusters among differentially expressed 30 HGRMDEGs in CRC

**Figure 5:**
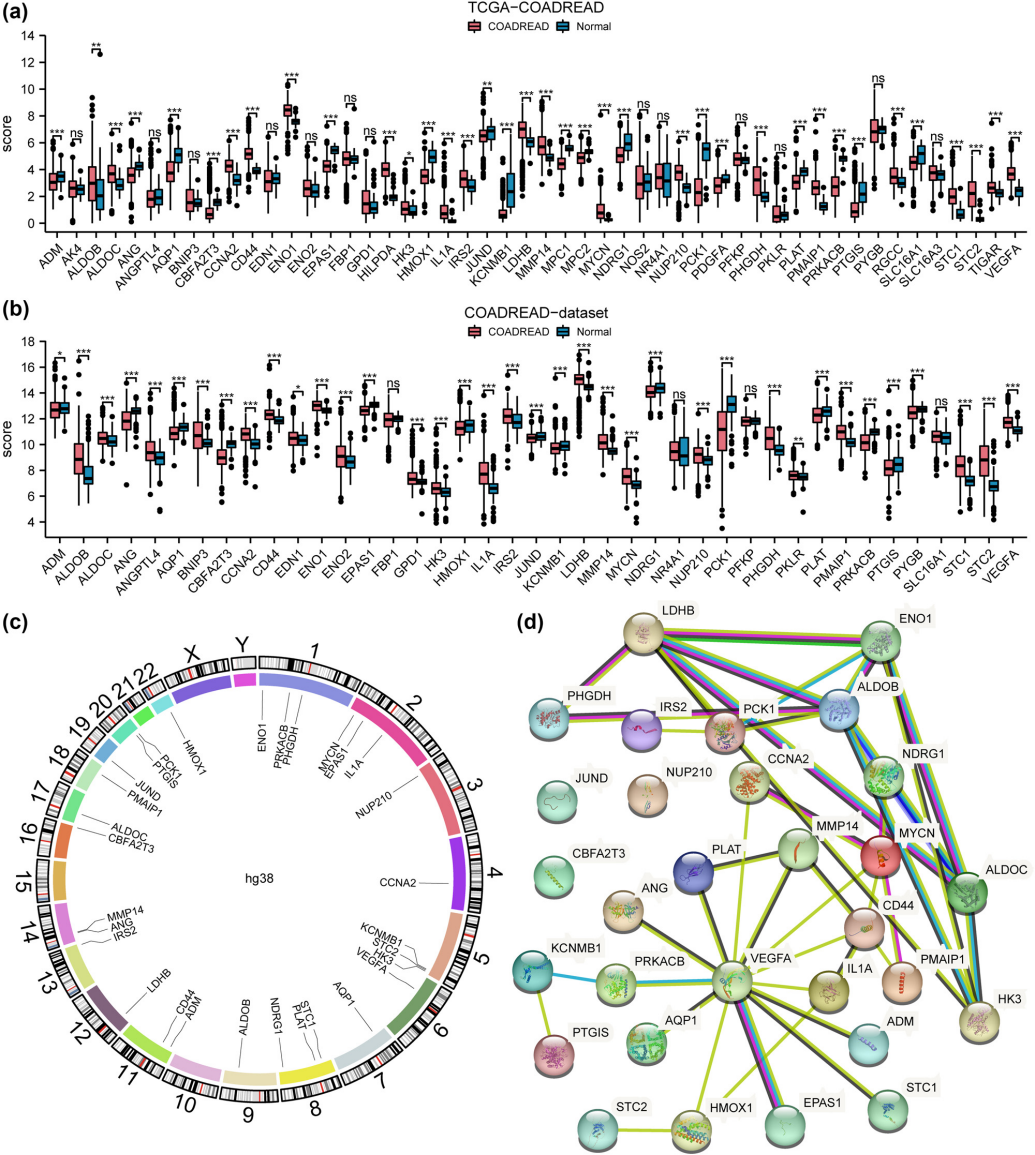
Chromosomal localization and PPI network analysis reveal functional clusters among differentially expressed 30 HGRMDEGs in CRC. The levels of HGRMDEGs in the TCGA-COADREAD (a) and COADREAD datasets (b). (c) Chromosomal localizations of HGRMDEGs. (d) PPI network of HGRMDEGs. NS: *P* ≥ 0.05, *P < 0.05, **P < 0.01, and ***P < 0.001.

We compared the expression levels of 50 HGRMDEGs (Table S4) between the cancer and normal groups in the COADREAD and TCGA-COADREAD datasets (Figure 5a and b). The analyses revealed 30 HGRMDEGs with consistent expression trend in both datasets, including *ADM*, *ALDOB*, *ALDOC*, *ANG*, *AQP1*, *CBFA2T3*, *CCNA2*, *CD44*, *ENO1*, *EPAS1*, *HK3*, *HMOX1*, *IL1A*, *IRS2*, *JUND*, *KCNMB1*, *LDHB*, *MMP14*, *MYCN*, *NDRG1*, *NUP210*, *PCK1*, *PHGDH*, *PLAT*, *PMAIP1*, *PRKACB*, *PTGIS*, *STC1*, *STC2*, and *VEGFA*. We further explored the chromosomal localization analysis of these 30 HGRMDEGs to observe their distributions. The chromosome localization map (Figure 5c) showed that these genes were primarily located on chromosomes 1, 2, 5, and 8, with each hosting three genes. The genes on the same chromosome might have functional connections. We then analyzed the PPI of these 30 HGRMDEGs in the STRING database by setting the required minimum interaction score to medium confidence (0.4). Subsequently, a PPI network comprising 27 genes was established and visualized using Cytoscape software (Figure 5d). Notably, at the lowest interaction score of 0.4, all genes interacted with at least one HGRMDEG, except CBFA2T3, JUND, and NUP210, which did not interact with other HGRMDEGs.

### 3.6 A 10-gene hypoxia–glycolysis signature predicts poor prognosis in CRC and unveils a core metabolic mechanism

**Figure 6:**
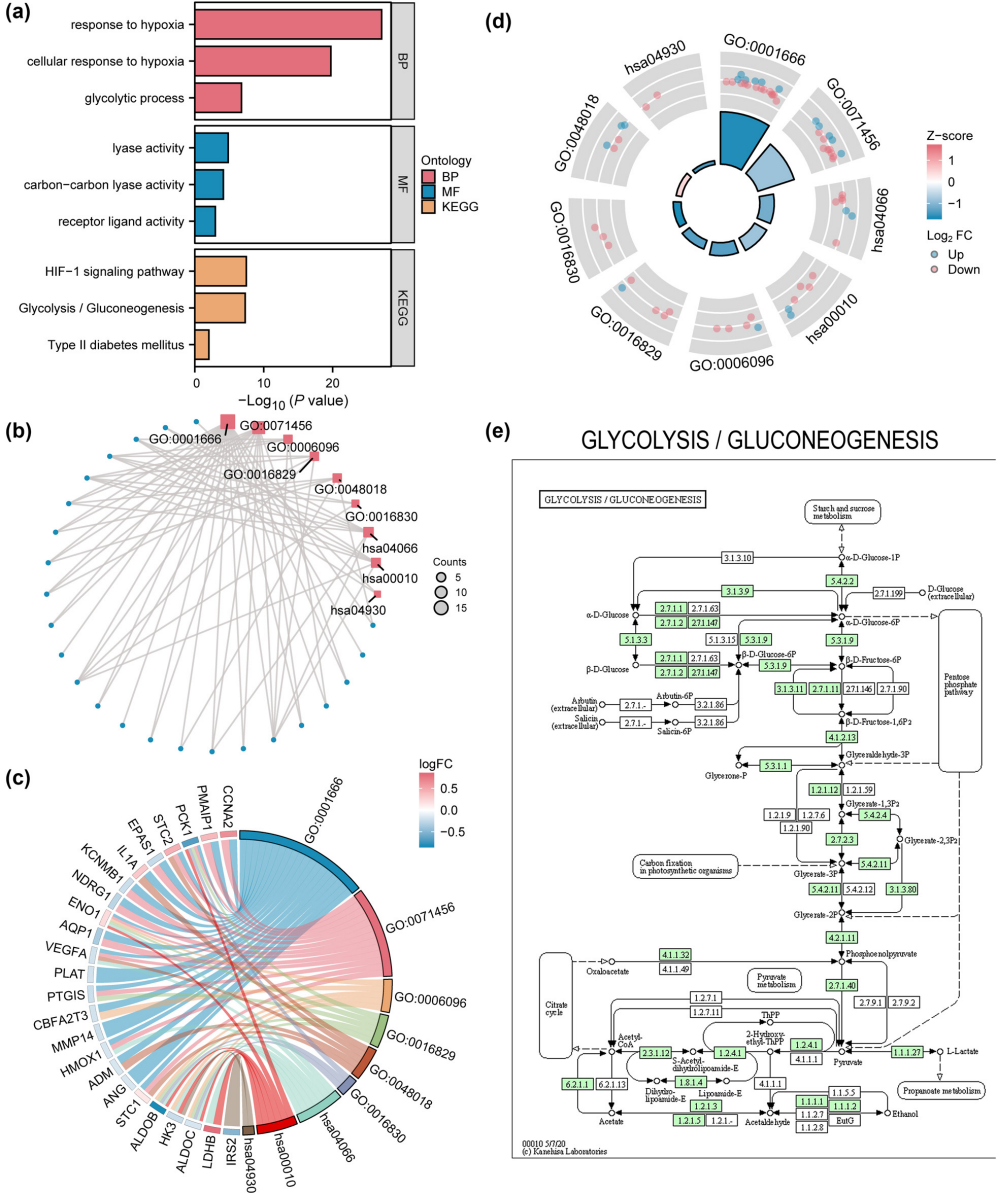
A 10-gene hypoxia-glycolysis signature predicts poor prognosis in CRC and unveils a core metabolic mechanism. Significantly enriched GO and KEGG terms of HGRMDEGs with bar graph (a) and circular network diagram (b). GO and KEGG enrichment analyses with logFC by chord diagram (c) and circle diagram (d). (e) The KEGG pathway diagram of Glycolysis/Gluconeogenesis (hsa00010). In (b), the blue dots and red squares represent specific genes and pathways, respectively. In (d), red and blue dots represent upregulated (logFC > 0) and down-regulated genes (logFC < 0), respectively. The screening criteria for GO and KEGG enrichment terms were *P* < 0.05 and FDR (*q* value) < 0.25.

GO enrichment (Table S5) unveiled 30 HGRMDEGs with predominant association with biological processes (BPs), such as response to hypoxia (GO: 0001666), cellular response to hypoxia (GO: 0071456), and glycolytic process (GO: 0006096) (Figure 6a), and molecular functions (MFs) including lyase activity (GO: 0016829), receptor-ligand activity (GO: 0048018) and carbon-carbon lyase activity (GO: 0016830) (Figure 6b). Further, KEGG enrichment analysis (Table S5) demonstrated their significant enrichment in the HIF-1α signaling pathway (hsa04066), glycolysis/gluconeogenesis (hsa00010), and type II diabetes mellitus (hsa04930) pathways. We further performed GO and KEGG enrichment analyses on these 30 HGRMDEGs based on the logFC values between the two mRNAsi groups in the TCGA-COADREAD dataset and calculated the corresponding *Z*-scores for each gene. The results are presented in the form of chord plots (Figure 6c) and circle plots (Figure 6d) with logFC. The circle plot shows that carbon-carbon cleavage enzyme activity (GO: 0016830) was significantly upregulated. The glycolysis/gluconeogenesis (hsa00010) pathway was selected to illustrate the KEGG pathway (Figure 6e).

### 3.7 The LASSO model stratifies CRC risk with survival prediction and accuracy

**Figure 7:**
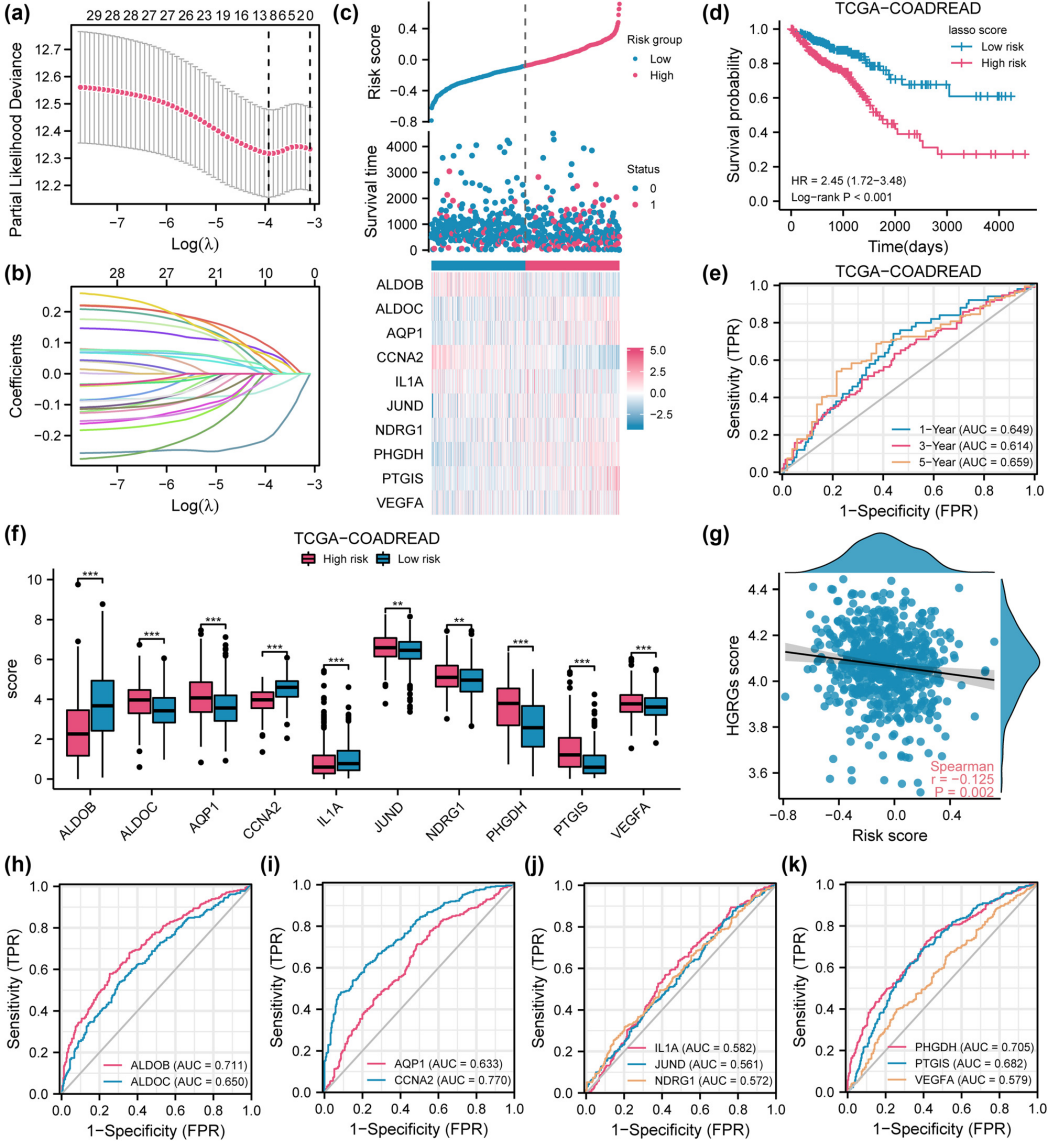
LASSO model stratifies CRC risk with survival prediction and accuracy. (a) Optimization parameter lambda determined by the LASSO regression model of HGRMDEGs. LASSO coefficient profile of the 10 HGRMDEGs (b) and risk factor plot (c) of the LASSO regression model. (d) Kaplan–Meier curves comparing the two risk groups in the TCGA-COADREAD dataset. (e) Time-dependent ROC curves illustrating the risk score for predicting COADREAD prognosis. (f) Comparison of the 10 HGRMDEGs levels between the two risk groups in the TCGA-COADREAD dataset. (g) Correlation between the risk score and the HGRG score of COADREAD patients. (h–k) The ROC curves of the 10 HGRMDEGs in the TCGA-COADREAD dataset. In the LASSO regression model plot (a), the *y*-axis represents the likelihood deviation, and the *x*-axis shows the log (λ), representing the penalty term in the LASSO regression. The numbers on the upper *x*-axis represent nonzero coefficients under each lambda. In the risk factor plot (c), red dots and blue dots represent the deceased and surviving patients, respectively. **P < 0.01 and ***P < 0.001.

To examine the prognostic value of the 30 HGRMDEGs (*ADM*, *ALDOB*, *ALDOC*, *ANG*, *AQP*, *CBFA2T3*, *CCNA2*, *CD44*, *ENO1*, *EPAS1*, *HK3*, *HMOX1*, *IL1A*, *IRS2*, *JUND*, *KCNMB1*, *LDHB*, *MMP14*, *MYCN*, *NDRG1*, *NUP210*, *PCK1*, *PHGDH*, *PLAT*, *PMAIP1*, *PRKACB*, *PTGIS*, *STC1*, *STC2*, *VEGFA*) in the TCGA-COADREAD dataset, we conducted a LASSO regression analysis and developed a prognostic model consisting of 10 HGRMDEGs (*ALDOB*, *ALDOC*, *AQP1*, *CCNA2*, *IL1A*, *JUND*, *NDRG1*, *PHGDH*, *PTGIS*, *VEGFA*) (Figure 7a and b). The risk factor plot (Figure 7c) visually depicts the risk groupings derived from the LASSO model. We categorized the cancer samples into two risk groups using the median risk score as the threshold. Subsequently, we plotted a prognostic Kaplan–Meier curve to compare survival outcomes between the two risk groups (Figure 7d). The analysis showed that the prognosis was significantly worse in the higher risk group (log-rank *P* < 0.001). We then plotted the prognostic time-dependent ROC curves for the risk scores (Figure 7e). The area under the curve (AUC) of the ROC curves for 1-, 3-, and 5-year survival was all greater than 0.6, suggesting that higher-risk scores were associated with worse prognosis in COADREAD. We further compared the levels of 10 HGRMDEGs in the LASSO prognostic model between the two groups (Figure 7f) and observed significant differences in the expression of these genes between the two groups (*P* < 0.01). To investigate the association between risk scores and HGRGs scores in COADREAD patients, we visualized the correlation between the two using a scatterplot (Figure 7g), which showed a significant (*P* < 0.05) but very weak (|*r*| < 0.3) linear correlation. In addition, ALDOB (AUC = 0.711), CCNA2 (AUC = 0.770), and PHGDH (AUC = 0.705) had some accuracy in predicting COADREAD high- and low-risk patients (Figure 7h–k).

### 3.8 Correlation and functional analysis reveal VEGFA dominance and mRNAsi-linked genes in CRC

**Figure 8:**
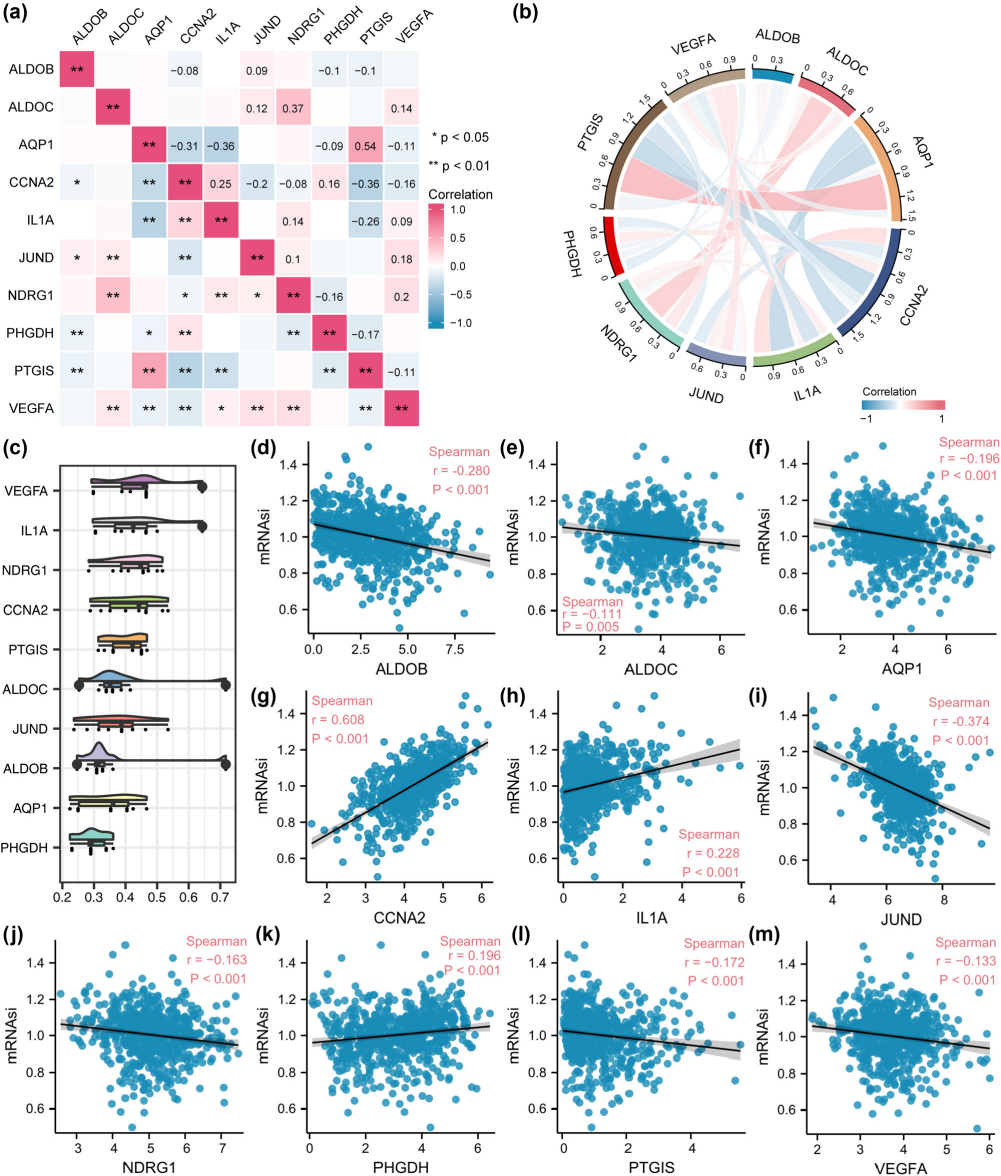
Correlation and functional analysis reveal VEGFA dominance and mRNAsi-linked genes in CRC. Correlation heatmap (a) and correlation chord diagram (b) displaying the correlation of HGRMDEGs in the TCGA-COADREAD dataset. (c) Box plot showing the functional similarity of HGRMDEGs. (d–m) Scatter plots exhibiting the association between HGRMDEGs and mRNAsi. *P < 0.05 and **P < 0.01. The absolute correlation coefficient (*r*) value in the scatter plots denotes a strong correlation if it is above 0.8, a moderate correlation if it is between 0.5 and 0.8, a weak correlation if it is between 0.3 and 0.5, and little or no correlation if it is below 0.3.

Based on the levels of 10 HGRMDEGs (*ALDOB*, *ALDOC*, *AQP1*, *CCNA2*, *IL1A*, *JUND*, *NDRG1*, *PHGDH*, *PTGIS*, *VEGFA*) in cancer samples in the TCGA-COADREAD dataset, we first investigated the correlation between them using the Spearman algorithm and presented the results in the form of correlation heatmaps (Figure 8a) and correlation chord plots (Figure 8b). The results showed a linear correlation of *ALDOC* with *NDRG1*, *AQP1* with *CCNA2*, *IL1A*, and *PTGIS*, and *CCNA2* with *PTGIS* (|*r*| > 0.3, *P* < 0.05). Subsequently, a functional similarity analysis to assess the similarities among the 10 HGRMDEGs (Figure 8c) revealed that VEGFA exhibited the highest functional similarity score. We further investigated the association of the levels of these ten genes with the corresponding mRNAsi in the cancer samples (Figure 8d–m) and showed that CCNA2 (*r* = 0.608, *P* < 0.001, Figure 8g) and PTGIS (*r* = −0.347, *P* < 0.001, Figure 8i) were significantly and linearly correlated with mRNAsi.

### 3.9 High-risk CRC genomic profiling links to immune evasion and ICB resistance

**Figure 9:**
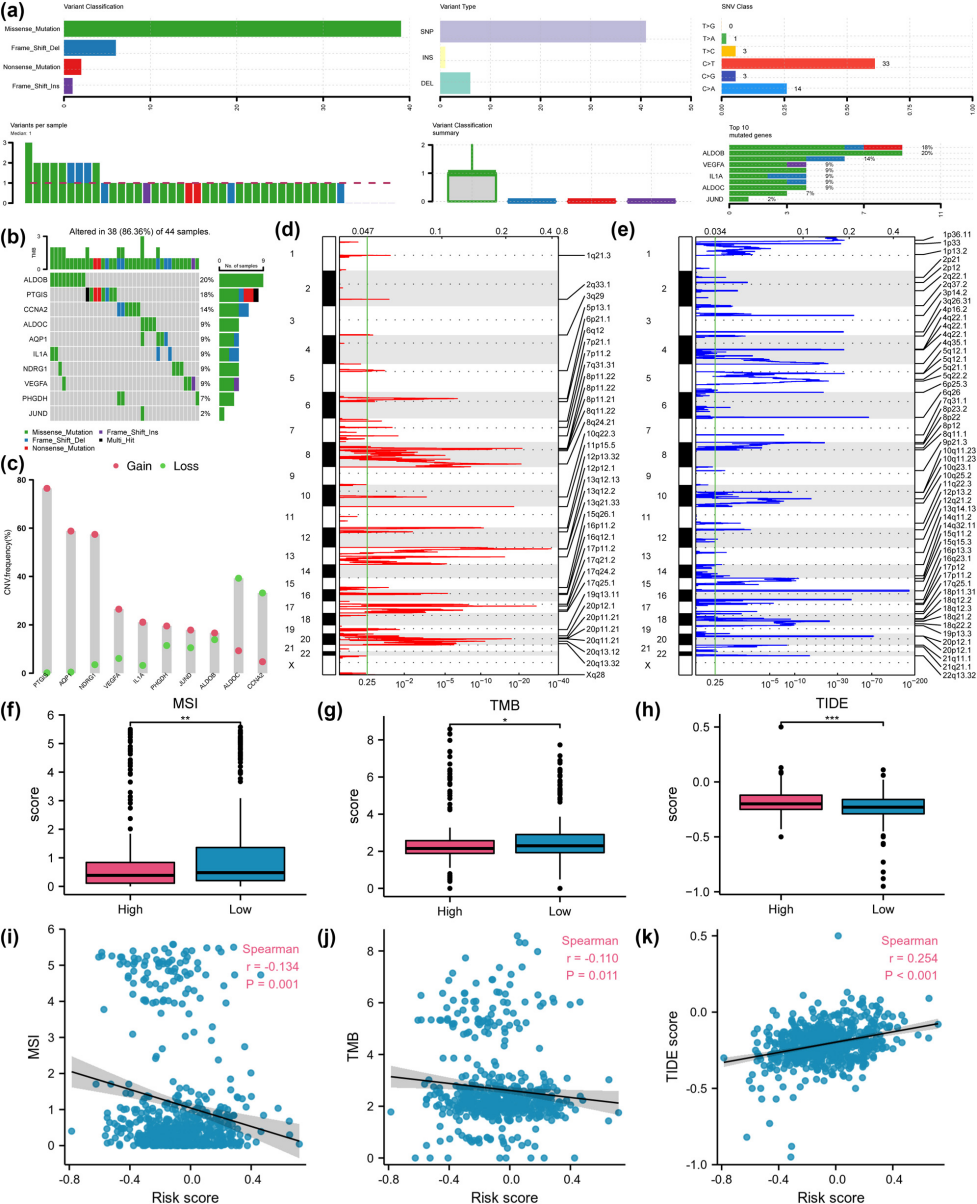
High-risk CRC genomic profiling links to immune evasion and ICB resistance. (a) Mutation type distributions of 10 HGRMDEGs in COADREAD patients. (b) Waterfall plot of somatic mutations. (c) CNV of 10 HGRMDEGs in COADREAD patients. (d) Genes with significant copy number amplification in COADREAD patients. (e) Genes with significant copy number deletion in COADREAD patients. Comparison of MSI (f), TMB (g), and TIDE score (h) between the two risk groups of COADREAD patients. Scatter plots showing the association of MSI (i), TMB (j), and TIDE score (k) with the risk score in COADREAD patients. *P < 0.05, **P < 0.01, and ***P < 0.001. In the scatter plot, the absolute correlation coefficient (*r*) > 0.8 indicates a strong correlation; 0.5–0.8 indicates a moderate correlation; 0.3–0.5 indicates a weak correlation, and <0.3 indicates little or no correlation.

Given that somatic genomic alterations (including mutations, CNVs, MSIs, and TMBs) are central mechanisms for cancer development, driving key pathways and influencing the immune microenvironment and therapeutic response, we further explored the somatic mutational profiles of 10 key HGRMDEGs and their association with risk models. The results showed that the TCGA-COADREAD dataset contains four types of somatic mutations: missense and nonsense substitutions and frame-shift deletions and insertions, with missense substitutions being the most prevalent (Figure 9a). Furthermore, the mutation types of the 10 HGRMDEGs in COADREAD patients primarily included SNPs along with some insertions and deletions (INS and DEL). Among SNVs, C > T was dominant, followed by C > A (Figure 8a). Among the 10 HGRMDEGs, *ALDOB* had the most somatic mutations, including missense, frame-shift deletion, and nonsense mutations (Figure 9b).

We also examined CNVs of the 10 HGRMDEGs in COADREAD patients from the TCGA-COADREAD dataset using GISTIC 2.0 (Figure 9c–e) and identified significant amplifications and deletions in the 10 HGRMDEGs among the COADREAD patient samples. Among these genes, *PTGIS*, *AQP1*, and *NDRG1* had the highest amplification frequency, while *ALDOC*, *CCNA2*, and *ALDOB* had the highest deletion frequency (Figure 9c).

Subsequently, we processed MSI and TMB data corresponding to COADREAD patients from the TCGA-COADREAD dataset, evaluated the TIDE scores using the TIDE algorithm, and generated grouped comparison charts (Figure 9f–h) and correlation scatter plots (Figure 9i–k) with respect to the corresponding risk scores. Our findings indicated statistically significant variations in MSI, TMB, and TIDE scores between the two risk groups (*P* < 0.05). Specifically, the high-risk group displayed lower MSI and TMB levels but higher TIDE scores. This signifies that the high-risk group is susceptible to immune escape and benefits less from immune checkpoint inhibitor therapy. Correlation scatter plots showed that MSI and TMB were weakly linearly associated with risk scores in COADREAD patients.

### 3.10 Gene-miRNA networks and consensus clustering identify prognostic subtypes in CRC

**Figure 10:**
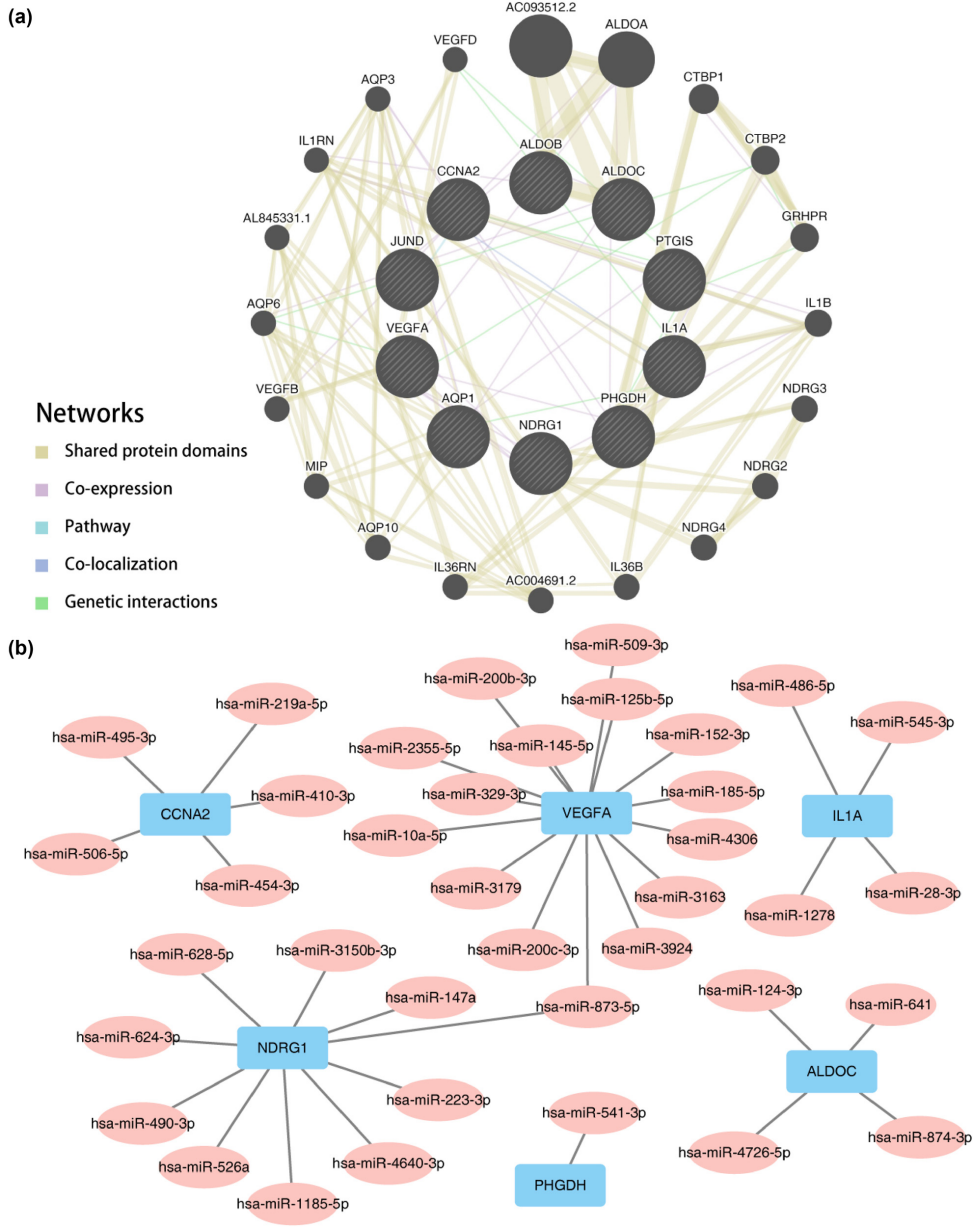
PPI network. (a) The PPI network of functionally similar genes related to the 10 HGRMDEGs. (b) The mRNA-miRNA regulatory network of the 10 HGRMDEGs, with blue rectangles representing mRNA and pink ellipses representing miRNA. The co-expression network (a) was generated using the GeneMANIA website. Black circles with white diagonal lines represent input RNAMRGs, while other black circles represent predicted functionally similar genes. Yellow-green lines represent shared protein domain relationships between genes; purple lines represent co-expression relationships between genes; light blue lines represent pathway-related connections between genes; dark blue lines represent co-localization relationships between genes; and green lines represent genetic interactions between genes.

We established an interaction network of functionally similar genes related to the 10 HGRMDEGs using the GeneMANIA website (Figure 10) to observe shared protein domains, co-expression, pathways, co-localization, and gene interactions. To identify miRNAs interacting with these 10 HGRMDEGs, mRNA–miRNA data from the ENCORI database were utilized. Subsequently, miRNAs were screened against more than three databases, and the mRNA–miRNA interaction network consisting of 6 mRNAs and 38 miRNAs was depicted using Cytoscape software (Figure 10) and Table S6.

**Figure 11:**
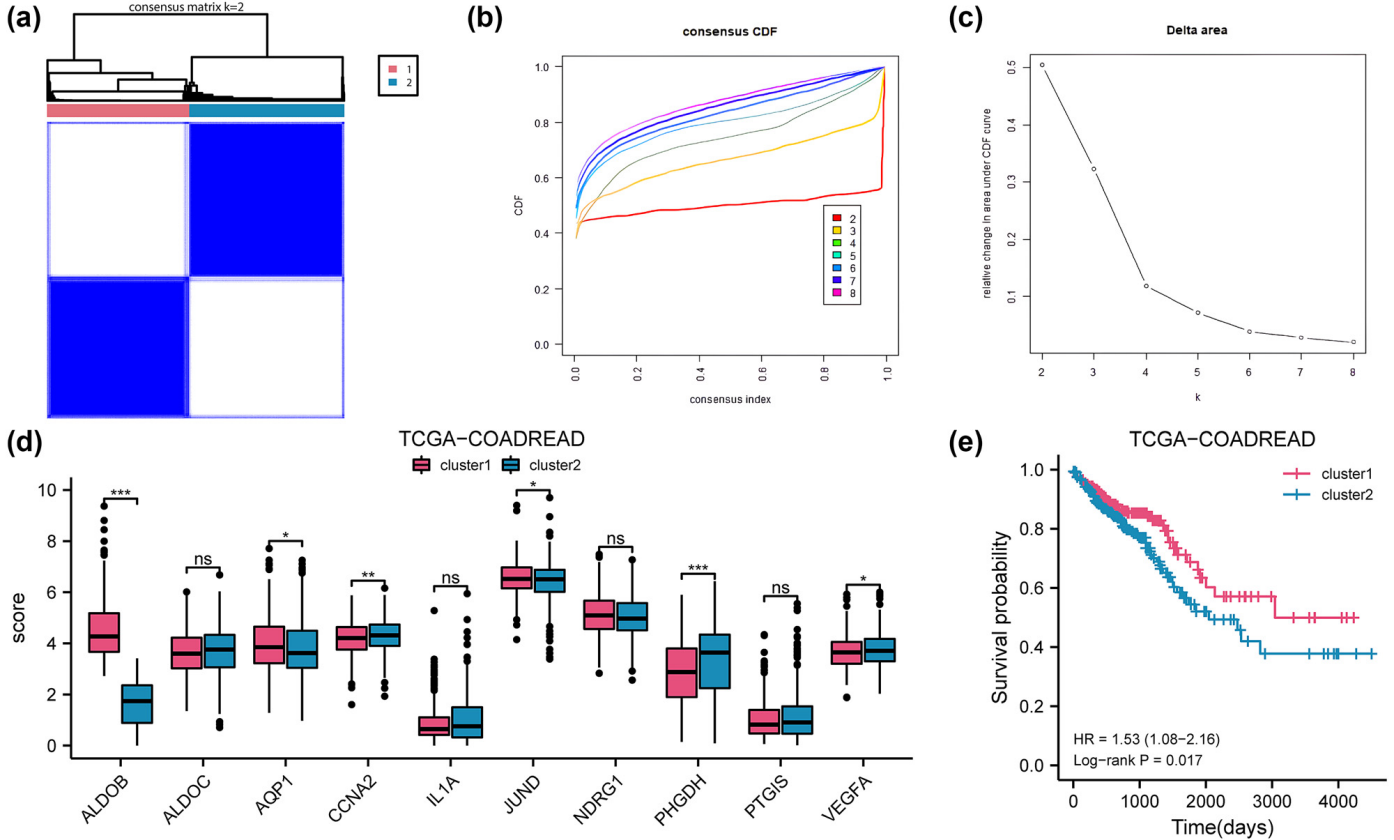
Consensus clustering identifies prognostic subtypes in CRC. (a) The COADREAD patients were divided into two clusters based on the consensus clustering matrix (*K* = 2). (b) Consensus clustering model with cumulative distribution function (CDF) for *k* values ranging from 2 to 9. (c) The delta area under CDF curves for *k* values ranging from 2 to 9. (d) The expression levels of the 10 HGRMDEGs between cluster 1 and cluster 2 in the TCGA-COADREAD dataset. (e) Kaplan–Meier curves between cluster 1 and cluster 2 in the TCGA-COADREAD dataset. ns: *P* ≥ 0.05, *P < 0.05, **P < 0.01, and ***P < 0.001.

To identify clinically significant molecular subtypes and to explore the association of the 10 HGRMDEG levels with COADREAD subtypes, we used consensus cluster analysis. The analysis categorized COADREAD disease into two subtypes: cluster 1 contained 308 samples and cluster 2 contained 336 samples (Figure 11a–c). In the TCGA-COADREAD dataset, 6 out of 10 HGRMDEGs showed significant expression differences between the two subtypes (*P* < 0.05, Figure 11d). The further constructed Kaplan–Meier survival curves showed that there was a significant difference in prognosis between the two groups (log-rank *P* = 0.017), with cluster 2 patients having a worse prognosis than cluster 1 (Figure 11e).

### 3.11 HGRG score stratification guides precision medication in CRC-high scoring patients have significantly increased sensitivity to 20 targeted agents

To explore the sensitivity of COADREAD patients to commonly used anticancer drugs in the TCGA-COADREAD dataset and to investigate potential treatment strategies for patients with different HGRG scores, we studied drug sensitivity using the GDSC database as a training set. We compared the IC50 of anticancer drugs in the two groups with different HGRG scores and used violin plots to show the top 20 drugs with significant differences in IC50 (Figure S2a–t). Among these 20 drugs, patients with higher HGRG scores had lower IC50 values compared to those with lower HGRG scores, suggesting that individuals with higher HGRG scores may be more sensitive to these drugs. This further emphasizes the importance of personalized treatment for cancer patients.

### 3.12 HGRG score and significant association with tumor microenvironmental characteristics

We investigated the expression profiles of cancer samples in the TCGA-COADREAD dataset, including stromal, immune, ESTIMATE, and tumor purity scores, as well as their distributions with respect to HGRGs scores, using the R package ESTIMATE. Our results showed that there were substantial differences (*P* < 0.001) in stroma (Figure S3a), immune (Figure S3b), ESTIMATE (Figure S3c), and tumor purity (Figure S3d) scores between the two groups of patients.

We further analyzed the association between HGRG scores and stromal, immune, ESTIMATE, and tumor purity scores of COADREAD patients and found that HGRG scores were weakly positively associated with stromal (*r* = 0.151, *P* < 0.001, Figure S3e), immune (*r* = 0.192, *P* < 0.001, Figure S3f), and ESTIMATE scores (*r* = 0.184, *P* < 0.001, Figure S3g) and weakly negatively correlated with tumor purity score (*r* = −0.184, *P* < 0.001, Figure S3h).

### 3.13 Characteristics of immune cell enrichment and key gene regulatory hubs in high HGRG-scoring groups

To examine differences in immune infiltration between the 2 HGRG score groups, we used multiple algorithms to calculate the abundance of 28 immune cell types in cancer patients from the TCGA-COADREAD dataset. We first applied the CIBERSORTx algorithm (Figure S4a) to visualize the distribution of immune cell types; subsequently, group comparisons were plotted by other algorithms (CIBERSORTx, MCPcounter, and ssGSEA) to demonstrate the changes in abundance between the two HGRG score groups (Figure S4b–d). The results showed that the high HGRG group presented significant enrichment in 25 out of 28 immune cell types (*P* < 0.05), suggesting the presence of extensive enhanced immune infiltration in the high HGRG group.

The findings exhibited that (1) when using the CIBERSORTx algorithm, 9 out of 22 immune cells demonstrated statistically significant differences (*P* < 0.05), 2) when using the MCPcounter algorithm, 8 out of 10 immune cells and stromal cells manifested significant differences (*P* < 0.05), 3) when with the ssGSEA algorithm, 25 out of 28 immune cells presented statistically significant differences (*P* < 0.05).

**Figure 12:**
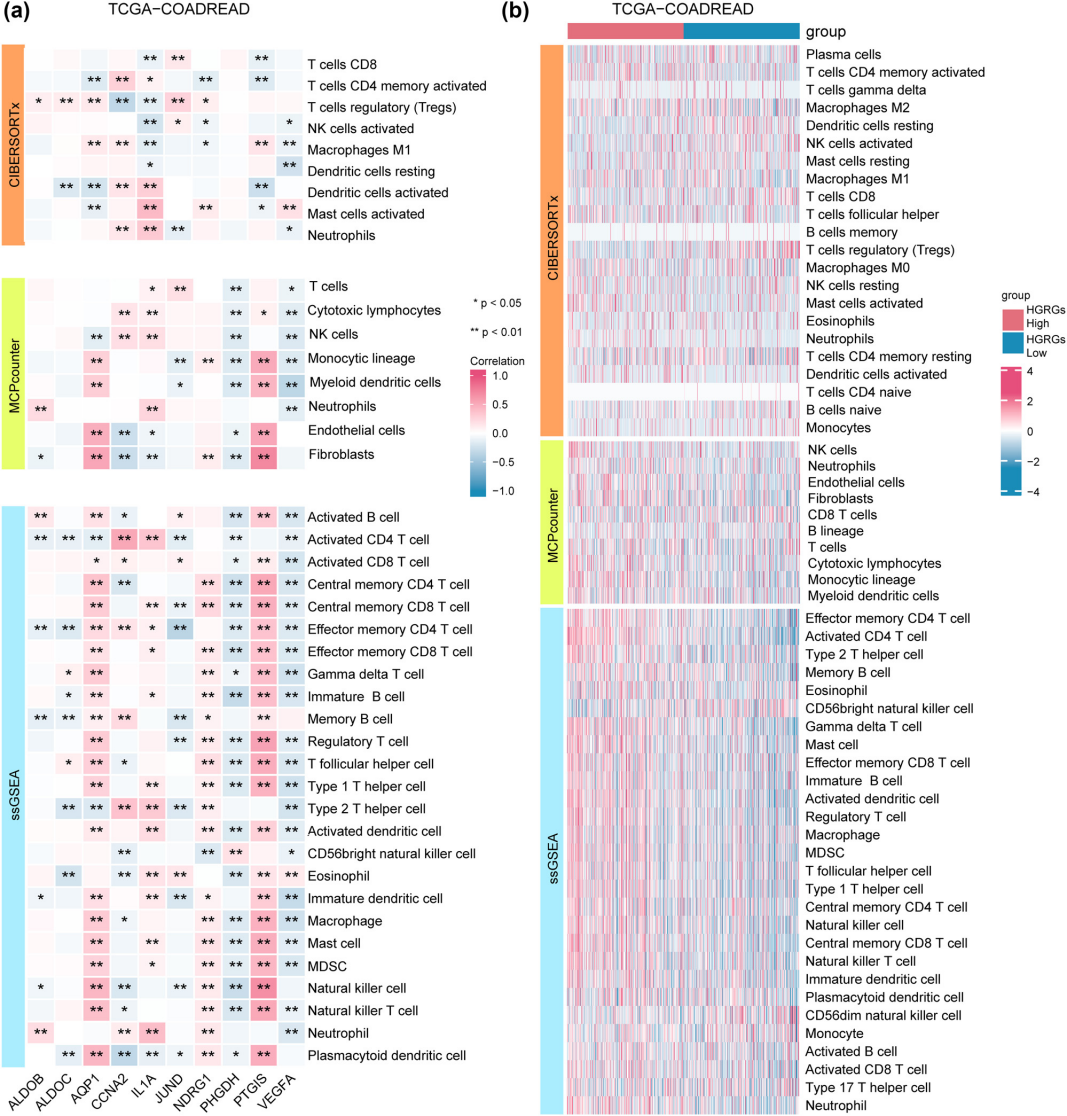
Characteristics of immune cell enrichment and key gene regulatory hubs in high HGRG scoring groups. (a) The associations between the 10 HGRMDEGs and the immune cell infiltration abundance under the CIBERSORTx algorithm, MCPcounter algorithm, and ssGSEA algorithm. (b) The infiltration abundance of all immune cells under the CIBERSORTx algorithm, MCPcounter algorithm, and ssGSEA algorithm in the two HGRGs score groups. *P < 0.05 and **P < 0.01.

We then selected immune cells with differential infiltration abundance (*P* < 0.05) between the two HGRG score groups to analyze their relationships with the 10 HGRMDEGs. The results (Figure 12a) showed that (1) under both MCPcounter and ssGSEA algorithms, immune cells exhibited positive linear correlations with *AQP1* and *PTGIS* immune cells but negative linear correlations with *PHGDH* and (2) immune cells exhibited a negative linear correlation with *VEGFA* under all three algorithms. We also plotted a complex heatmap to show the infiltration abundance of all immune cells in the two HGRG score groups under the three algorithms (Figure 12b). Notably, the high HGRGsscore group presented significantly increased immune cell infiltration abundance under both MCPcounter and ssGSEA algorithms.

### 3.14 HGRMDEGs identified as prognostic biomarkers linking TNM staging to clinical outcomes in CRC

**Figure 13:**
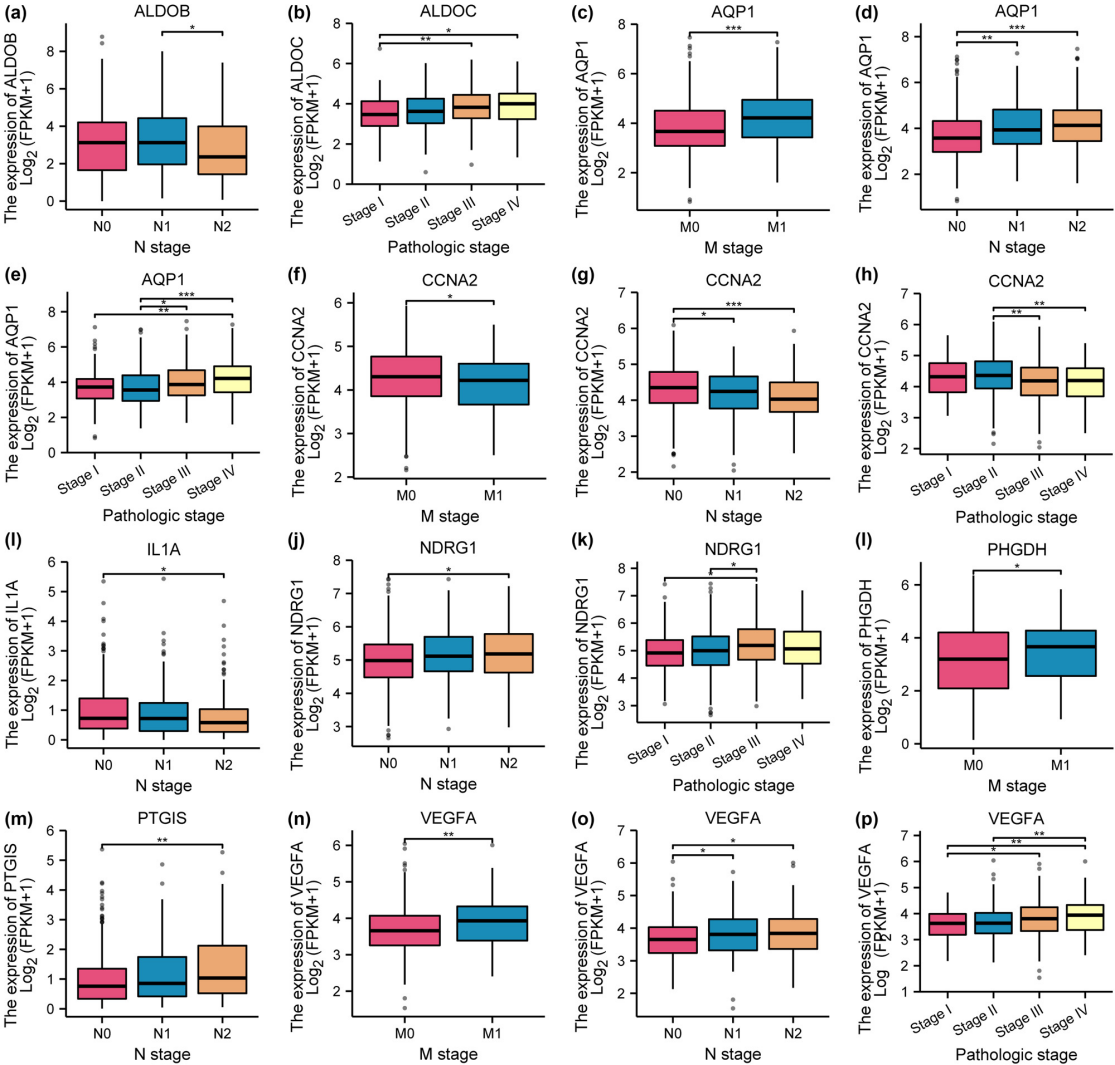
HGRMDEGs identified as prognostic biomarkers linking TNM staging to clinical outcomes in CRC. (a–p) The correlations of HGRMDEGs with N, M, and pathological stages. *P < 0.05, **P < 0.01, and ***P < 0.001.

We further comparatively analyzed the levels of the 10 HGRMDEGs in patients at different clinical substages, including T1, T2, T3, T4, N0, N1, N2, N3, M0, M1, and pathological stages I, II, III, and IV. The outcomes displayed a potential association between N stage subtype and the genes ALDOB (Figure 13a), AQP1 (Figure 13d), CCNA2 (Figure 13g), IL1A (Figure 13i), NDRG1 (Figure 13j), PHGDH (Figure 13m), and VEGFA (Figure 13o). Furthermore, the expression of genes AQP1 (Figure 13c), CCNA2 (Figure 13f), PHGDH (Figure 13m), and VEGFA (Figure 13n) showed a potential association with M stage subtype. Lastly, the genes ALDOC (Figure 13b), AQP1 (Figure 13e), CCNA2 (Figure 13h), NDRG1 (Figure 13k), and VEGFA (Figure 13p) exhibited a potential association with clinical pathologic stage.

### 3.15 The prediction model for CRC shows high prediction accuracy and high net benefit

**Figure 14:**
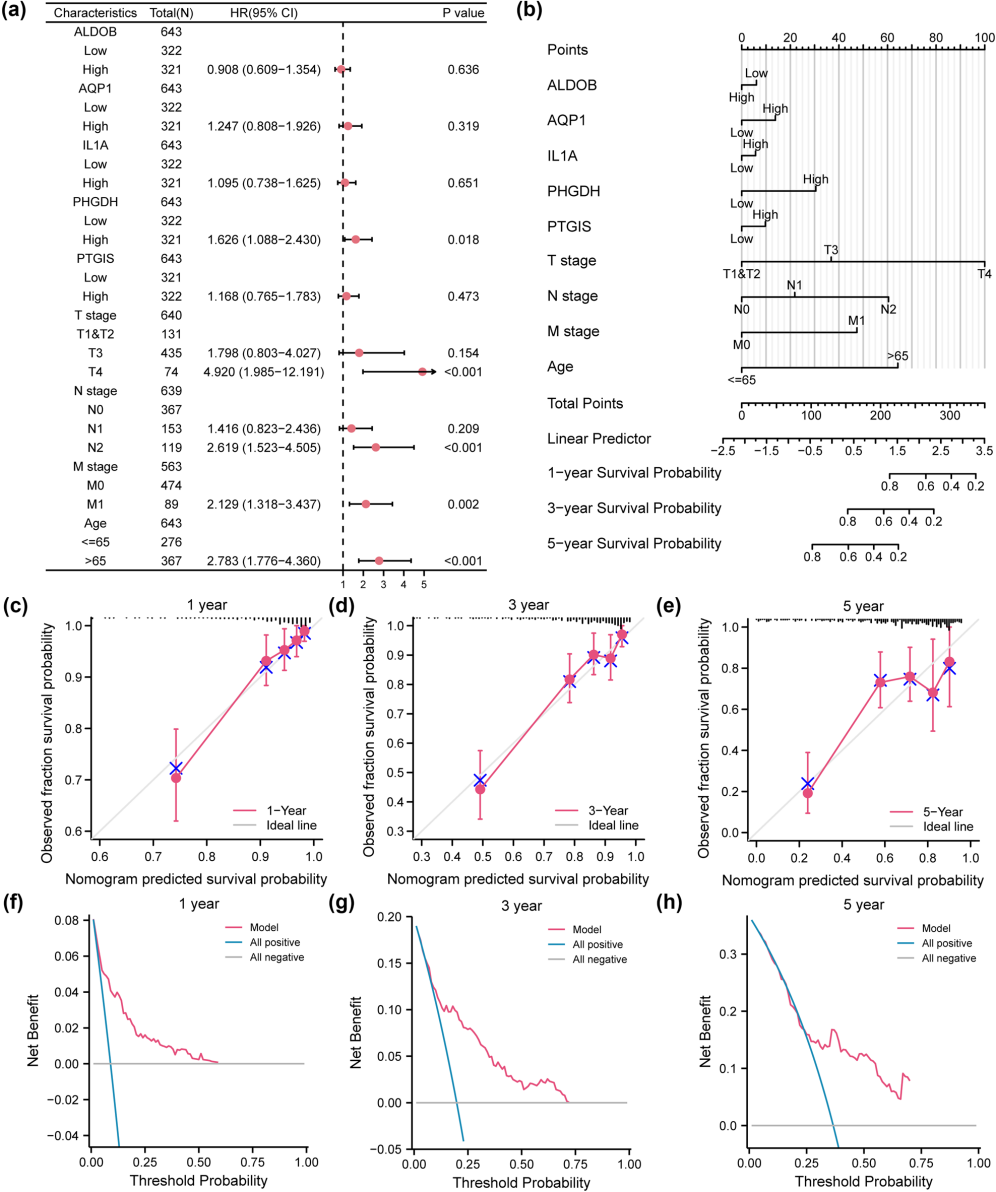
The prediction model for CRC shows high prediction accuracy and high net benefit. The forest plot (a) and the nomogram (b) of the multivariate Cox regression model. The calibration curves of the nomogram at 1-year (c), 3-year (d), and 5-year (e) intervals. DCA plots of the nomograms at 1-year (f), 3-year (g), and 5-year (h) intervals. The *x*-axis in the DCA plot represents the threshold probability, while the *y*-axis represents the net benefit.

At last, we analyzed the clinical traits of COADREAD patients in the TCGA-COADREAD dataset (Table 1) and executed univariate Cox regression analyses to examine their association with the 10 HGRMDEGs. Variables with *P* < 0.1, including T, N, M, and pathological stages, as well as age and BMI, were subjected to multivariate Cox regression analysis. The optimal variables, including T, N, and M stages, as well as age, were further identified with stepwise regression and chosen to create the multivariate Cox regression model (Table S7). The findings were visualized in a forest plot (Figure 14a). Furthermore, we performed nomogram analyses to judge the predictive value of the genes and clinical traits included in the multivariate Cox regression model (Figure 14b). Additionally, we generated calibration curves for the 1-, 3-, and 5-year survival (Figure 14c–e) and demonstrated that the red lines corresponding to the 1- and 3-year survival were near the ideal gray line, suggesting strong predictive performance of the model. Lastly, we evaluated the clinical application of the multivariate Cox regression model using DCA (Figure 14f–h). The results revealed that the red lines representing the model were significantly distant from both the blue lines (all positive) and the gray line (all negative), indicating its high accuracy.

**Table 1:** Clinicopathological features of patients with CRC

Characteristic	Levels	Overall
*N*		644
T stage, *n* (%)	T1	20 (3.1%)
	T2	111 (17.3%)
	T3	436 (68%)
	T4	74 (11.5%)
N stage, *n* (%)	N0	368 (57.5%)
	N1	153 (23.9%)
	N2	119 (18.6%)
M stage, *n* (%)	M0	475 (84.2%)
	M1	89 (15.8%)
Pathologic stage, *n* (%)	Stage I	111 (17.8%)
	Stage II	238 (38.2%)
	Stage III	184 (29.5%)
	Stage IV	90 (14.4%)
Gender, *n* (%)	Female	301 (46.7%)
	Male	343 (53.3%)
Age, *n* (%)	≤65	276 (42.9%)
	>65	368 (57.1%)
BMI, *n* (%)	<25	107 (32.5%)
	≥25	222 (67.5%)
OS event, *n* (%)	Alive	515 (80%)
	Dead	129 (20%)
Age, median (IQR)		68 (58, 76)

## 4 Discussion

As a global, highly prevalent malignant tumour, the molecular heterogeneity of CRC has led to the insufficient predictive efficacy of existing biomarkers, which restricts the development of precision diagnosis and treatment. In this study, we broke through the limitations of a single molecular dimension and constructed a 10-gene prognostic model and disease subtype classification system through mRNAsi, hypoxia–glycolysis-related genes, and multi-omics analysis (DEG/WGCNA/LASSO). The key findings indicated that (1) patients in the high-risk group had significantly lower survival and limited response to immunotherapy; (2) subtype two (Cluster2) identified by consensus clustering had a worse prognosis; and (3) a column-line graph model based on TNM stage-age-core genes (C-index = 0.76–0.79) and DCA validation provided a quantitative tool for clinical decision-making. This multimodal analysis framework provides a new paradigm for molecular typing and individualized treatment of CRC.

The LASSO risk score model consists of ten genes: *ALDOB*, *ALDOC*, *AQP1*, *CCNA2*, *IL1A*, *JUND*, *NDRG1*, *PHGDH*, *PTGIS*, and *VEGFA*. As far as we know, both *ALDOB* and *ALDOC* belong to the aldolase family. Aldolase, as the fourth enzyme of the glycolysis pathway, is vital in glycolysis and gluconeogenesis. Previous explorations have revealed a correlation between high *ALDOB* levels and unfavorable survival outcomes for CRC patients. Additionally, increased *ALDOB* expression serves as a pivotal trigger for metabolic reprogramming [64,65]. *ALDOC* overexpression acts as an independent prognostic factor for CRC patients. Moreover, *ALDOC* downregulation inhibits cell proliferation, migration, and spheroid formation [66]. *AQP1* belongs to the aquaporin family and is responsible for the rapid passive transport of water across biological membranes. It has been shown that *AQP9* promotes cancer cell invasion and motility through the AKT signaling pathway [67]. Similarly, *AQP1* expression in CRC cells may enhance their ability to invade surrounding tissues and enter the bloodstream, thereby promoting spread to distal organs such as the liver. *AQP1* level is a poor prognostic factor for advanced colon cancer [68]. *CCNA2*, a vital member of the conserved cyclin family and referred to as cyclin A2, is critical in regulating the cell cycle. Additionally, *CCNA2* demonstrates a strong association with the survival rate of CRC patients. Silencing *CCNA2* suppresses CRC cell proliferation and induces apoptosis [69]. IL1A encodes IL-1α, which belongs to the IL-1 protein cluster, serves as a pro-inflammatory factor, and is considered a potential prognostic biomarker related to T-cell infiltration in CRC patients [70]. *JUND*, an AP-1 transcription factor, regulates cell differentiation, proliferation, and apoptosis [71]. *NDRG1* is involved in stress response and many BPs, such as hypoxia, cell proliferation, lipid metabolism, and chemotherapy resistance [72]. *NDRG1* downregulation and TNM stage are important independent factors affecting the OS of CRC patients [73]. Downregulation of *NDRG1* might promote epithelial–mesenchymal transition (EMT) progression in CRC through the NF-κB signaling [74]. PHGDH is an essential enzyme in synthesizing serine, a metabolic substrate for producing NADPH and glycine [75]. PHGDH-mediated serine metabolism promotes DNA hypermethylation in CRC stem cells by maintaining redox homeostasis and providing single-carbon units [76]. Inhibitors targeting PHGDH, such as NCT-503, have shown potential in cancer therapy. NCT-503 effectively inhibits PHGDH activity, thereby blocking the serine synthesis pathway, which in turn affects cancer cell growth and survival [77]. *PHGDH* overexpression is linked to the TNM stage and tumor size and independently predicts a poor prognosis in CRC patients [78]. PTGIS is an enzyme that converts prostaglandin H2 into prostaglandin I2 (PGI2). In CRC, PGE2 promotes tumor metastasis by upregulating miR-675-5p, a process that involves the regulation of p53 expression [79]. Furthermore, PGE2 regulates cell proliferation and signaling in CRC cells by inhibiting the expression of TIG1 and GRK5 and affecting the β-linker protein/TCF and cAMP signaling pathways [80]. Its expression is related to tumor growth and progression [81]. *PTGIS* overexpression is closely linked to liver metastasis and predicts a poor prognosis in colon cancer patients [82]. *VEGFA* is in mediating tumor angiogenesis, with its expression being controlled by oncogenes, diverse growth factors, and hypoxia. Nevertheless, the influence of *JUND* on the prognosis among CRC patients remains unexplored, and its effects on CRC patient prognosis have not been well explored. Therefore, from the initial ensemble of 273 HGRGs, a 10-gene prognostic model was constructed through multi-step screening such as DEG intersection, WGCNA modular association, and LASSO regression. The core advantages of this model are as follows: (1) pathway synergy can be captured, and the selected genes (e.g., glycolysis-related ALDOB, ALDOC, PHGDH, hypoxia-responsive VEGFA, and NDRG1) together constitute the core network of hypoxia-glycolysis-proliferation, avoiding single-gene misclassification. (2) Predictive robustness is improved, with multigene combinations significantly improving prognostic stratification (time-dependent ROC AUC > 0.6) and risk prediction accuracy (C-index 0.76–0.79) over single markers. (3) Regulatory hubs are revealed, and PPI network and functional analyses show that the 10 genes form a tight functional module with VEGFA and other hubs, providing a basis for multi-target combinations for co-targeted therapies.

The results obtained from GSEA indicated significant enrichment of all DEGs between the two mRNAsi groups in various pathways, including the cellular response to hypoxia, glycolysis, as well as Notch, JAK-STAT, Wnt, PI3K-AKT, MAPK, Hedgehog, and FcεRI-mediated NF-κB signaling pathways. GO analysis unveiled that the 30 HGRMDEGs were enriched in BPs related to response to hypoxia, cellular response to hypoxia, and glycolysis, as well as MFs involving lyase activity, receptor–ligand activity, and carbon–carbon lyase activity. The KEGG analysis of the 30 HGRMDEGs mainly revealed enrichment in the HIF-1α signaling pathway, glycolysis/gluconeogenesis, and type II diabetes mellitus pathway. Most of the enriched terms were associated with cancer hallmarks, including CSCs, hypoxia, and glycolysis. Wnt/β-catenin, Notch, and hypoxia signaling pathways are crucial in regulating CSC self-renewal and participating in tumorigenesis [83]. In addition, the WNT pathway is connected to the poor prognosis of CRC [84]. The PI3K-AKT pathway is vital in maintaining CSCs in multiple cancers, including CRC, promoting CSCs’ proliferation, migration, EMT, and invasion [85]. The AKT/mTOR/HIF-1α signaling pathway is crucial in promoting glycolysis and lactation, thus contributing to the “metabolic reprogramming” of cancer cells [86]. Epidemiological studies have established that type 2 diabetes mellitus (T2DM) is a risk factor for CRC [87]. Interestingly, our KEGG enrichment results included T2DM pathways, suggesting a potential shared risk factor between T2DM and CRC.

The multi-omics analysis in this study not only revealed the functions of key genes but also provided an in-depth portrayal of the tumor microenvironment shaped by them. Comprehensive analysis of the tumor microenvironment (including immuno-score, stroma-score, ESTIMATE-score, tumor purity, immune-cell infiltration as assessed by multiple algorithms, and the TIDE-score) showed that the high-risk-score group (based on the 10-gene signature) and/or high HGRG score groups exhibited significant alterations in tumor microenvironmental characteristics. Specifically, these groups were characterized by enhanced immunosuppression: e.g., ALDOB overexpression may inhibit CD8^+^ T-cell function through the WNT/PD-L1 axis [88], and IL1A promotes TAM-mediated immunosuppressive microenvironment formation [89]. High expression of the key gene VEGFA drives significant angiogenesis, which has been shown to be a major driver of tumor angiogenesis in the high-scoring group in animal models [90]. Combined CIBERSORTx, MCPcounter, and ssGSEA algorithm evaluations found that while the high-risk/high-HGRG group showed higher overall immune cell infiltration abundance (especially under ssGSEA and MCPcounter evaluations), this infiltration was accompanied by specific cellular subset disproportionality and altered functional status. MSI and TMB levels were significantly lower in the high-risk group compared to the low-risk group, while the TIDE score, which reflects immune escape potential, was significantly higher. Higher HGRG scores were associated with higher matrix scores, immune scores, and ESTIMATE scores, accompanied by reduced tumor purity. The synergistic effect of hypoxia-glycolysis-dryness features (characterized by HGRG scores and 10-gene risk scores) shapes a tumor microenvironment with immunosuppressive, angiogenically active, matrix-enriched features and a high potential for immune escape, which may be one of the core drivers of poor prognosis in high-risk patients and directly affects their response to immunotherapy. Therefore, risk scores derived from the analysis of 10 HGRGs can effectively identify high-risk patients with the aforementioned unfavorable tumor microenvironmental features and help select the most appropriate immunotherapy candidates.

The multivariate Cox regression model established using stepwise regression by incorporating *ALDOB*, *AQP1*, *IL1A*, *PHGDH*, and *PTGIS*, TNM stage, and age indicated that *PHGDH* could be an independent prognostic factor for CRC, in line with a previous report with a smaller sample size. Notably, our study, for the first time, identified a significant correlation between *PHGDH* and age. To enhance its clinical applicability, we established a prognostic nomogram by integrating *ALDOB*, *AQP1*, *IL1A*, *PHGDH*, and *PTGIS* with various clinicopathological features. Its accuracy was validated in the TCGA dataset, revealing a good predictive power. Notably, this is the first prognostic nomogram based on mRNAsi-related HGRGs, and these five important genes have yet to be included in other predictive models for cancers, including CRC. The utilization of this model could aid in molecular typing and screening of differential patient subgroups, ultimately optimizing personalized therapy.

Despite the valuable insights, this study had certain limitations. First, in terms of data sources, although multi-omics data (e.g., GEO and TCGA) have been integrated, the samples are mainly derived from Western populations, especially the TCGA database, which has more than 85% of Western populations. This data heterogeneity may limit the generalisability and accuracy of the constructed model in Asian populations. Second, in terms of computational drug sensitivity prediction, the pRophetic algorithm integrates GDSC and TCGA data to predict drug sensitivity in CRC and identify potential therapeutic clues for people with high HGRG scores. However, the model has three limitations: the biological level does not incorporate tumor microenvironment components (e.g., fibroblasts, Treg cells, tumor-associated macrophages) and dynamic interactions, the pharmacological mechanism only reflects *in vitro* cellular effects but ignores *in vivo* pharmacokinetics, and the heterogeneity is not sufficiently characterized to recapitulate the intra-tumor genetic/spatial heterogeneity. The performance of the algorithm is limited by the feature matching between the training set and the test set, and lacks prospective validation with clinical efficacy indexes. Therefore, the current drug sensitivity analysis should be regarded as an exploratory hypothesis to guide subsequent studies, and in the future, it should be deepened through the functional validation of patient-derived organoid/xenograft model, biomarker-based clinical drug tracking, and mechanistic studies (e.g., analysis of the association between drug targets and metabolic pathways), in order to provide a reliable basis for translational decision-making in the clinic. Furthermore, in terms of mechanistic studies, the functional regulatory networks of key genes (e.g., PHGDH and VEGFA) rely mainly on bioinformatics inference, while functional validation by gene editing (e.g., CRISPR) or organoid models is lacking. This lack of experimental validation makes the understanding of gene function insufficiently deep and accurate. Finally, in terms of the clinical application of the prognostic model, although the column-line diagram model has been validated by DCA, the supporting test kits (e.g., NGS panel) have not yet been developed, which limits the on-the-ground application and promotion of the model in clinical practice. In summary, the current study faces certain limitations in terms of data sources, computational drug sensitivity prediction, mechanism study, and clinical application of the prognostic model, which need to be further explored and addressed in future studies. Our future research interests include multi-centre cohort validation, organoid drug screening, functional genome analysis, and development of clinical translational tools. We plan to incorporate more than 2,000 samples from multiple centers in Asia to correct for population variation, analyze the tumour microenvironment using single-cell transcriptome technology, and develop race-adaptive algorithms. Meanwhile, the CRC organoid library is established to verify drug synergy, detect serine metabolism, and screen drug resistance genes. In terms of the functional genome, we validate the PHGDH regulatory mechanism, locate HGRG gene expression, and analyze the PHGDH transcriptional network. Finally, a portable gene testing kit and AI decision-making system are developed to prospectively validate the guiding value for chemotherapy regimen selection.

## 5 Conclusion

By integrating multi-omics data and systems biology analysis, this study deeply analyzed the interaction network between stemness characteristics and hypoxia–glycolysis pathway in CRC, and successfully established a molecular typing and prognosis prediction system with clinical translational value. The main conclusions include: the identification of metabolic-microenvironmental regulatory axes centered on PHGDH and VEGFA; the classification of CRC into two molecular subtypes with different therapeutic sensitivities, which provides a potential strategy for targeted metabolism in combination with antivascular therapies; and the construction of a columnar graph model that significantly optimizes prognosis prediction of patients with CRC, which is particularly valuable in individualized survival assessment of patients with advanced disease.

## Abbreviations

AUC: area under the curve
BP: biological process
CDF: cumulative distribution function
CNV: copy number variation
COADREAD: colon adenocarcinoma/rectal adenocarcinoma
DEG: differentially expressed gene
GDSC: Genomics of Drug Sensitivity in Cancer
GEO: Gene Expression Omnibus
GO: Gene Ontology
GSEA: gene set enrichment analysis
HGRGs: hypoxia and glycolysis-related genes
HGRMDEGs: hypoxia and glycolysis-related module DEGs
KEGG: Kyoto Encyclopedia of Genes and Genomes
LASSO: Least Absolute Shrinkage and Selection Operator
MF: molecular function
mRNAsi: mRNA stemness index
MSI: microsatellite instability
PCA: principal component analysis
PPI: protein–protein interaction
ROC: receiver operating characteristic curve
SNP: single-nucleotide polymorphism
ssGSEA: single-sample GSEA
DCA: decision curve analysis
TCGA: The Cancer Genome Atlas
TIDE: Tumor Immune Dysfunction and Exclusion
TMB: tumor mutational burden
WGCNA: weighted gene co-expression network analysis

## Supplementary Material

Supplementary Figure

Supplementary Table 1

Supplementary Table 2
