# Supplementary material for: A machine learning-based prognostic model integrating mRNA stemness index, hypoxia, and glycolysis‑related biomarkers for colorectal cancer
Source: Open Med (Wars). 2025 Sep 15;20(1):20251247. doi: 10.1515/med-2025-1247 (PMC12452079; doi:10.1515/med-2025-1247)
Supplement: Supplementary Figure [file med-2025-1247-sm.pdf]

Supplementary material

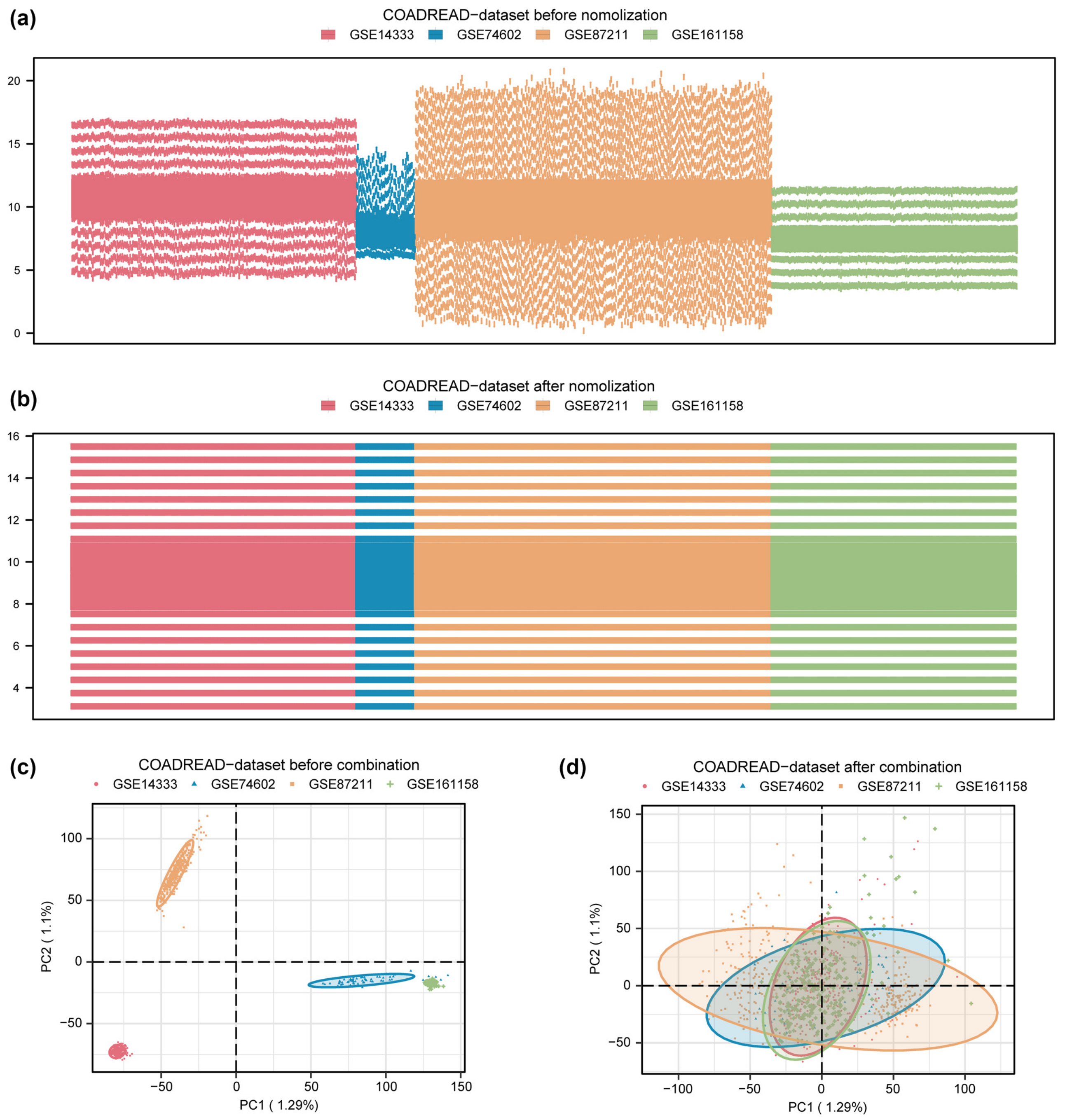

Figure S1: The distribution and PCA plots of COADREAD dataset pre- and post-merging. (a) and (b). The distribution boxplots of samples in pre- (a) and post- (b) integration COADREAD dataset. (c) and (d). The PCA plots of samples in pre- (c) and post- (d) integration COADREAD dataset.

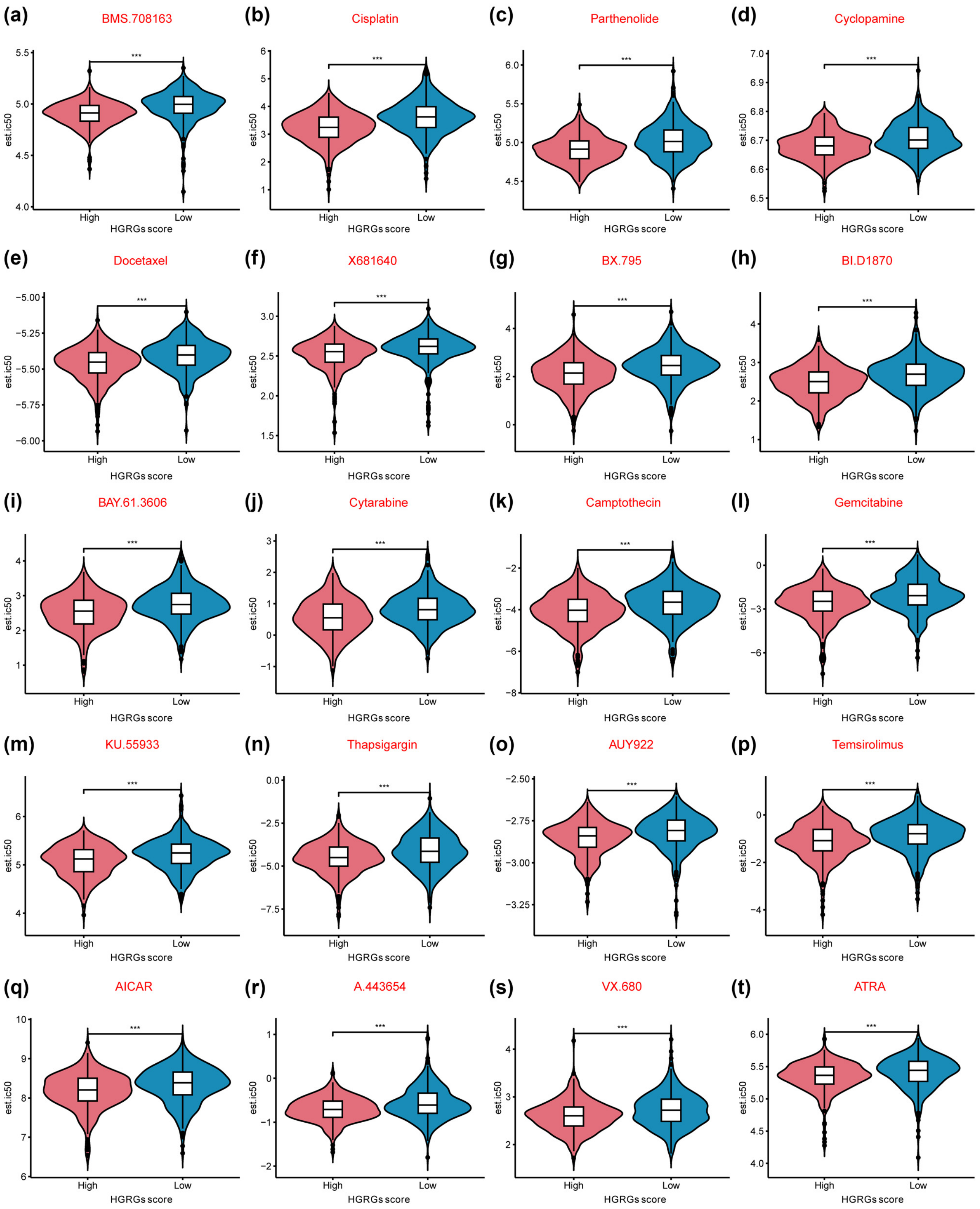

Figure S2: Drug sensitivity analysis. (a)–(t) Grouped comparison graphs of sensitivity of patients with high and low HGRGs scores in the TCGA-COADREAD dataset to drugs BMS.708163 (a), cisplatin (b), parthenolide (c), cyclopamine (d), docetaxel (e), X681640 (f), BX.795 (g), BI.D1870 (h), BAY.61.3606 (i), cytarabine (j), camptothecin (k), gemcitabine (l), KU.55933 (m), thapsigargin (n), AUY922 (o), temsirolimus (p), AICAR (q), A.443654 (r), VX.680 (s), and ATRA (t) based on data from the GDSC database.

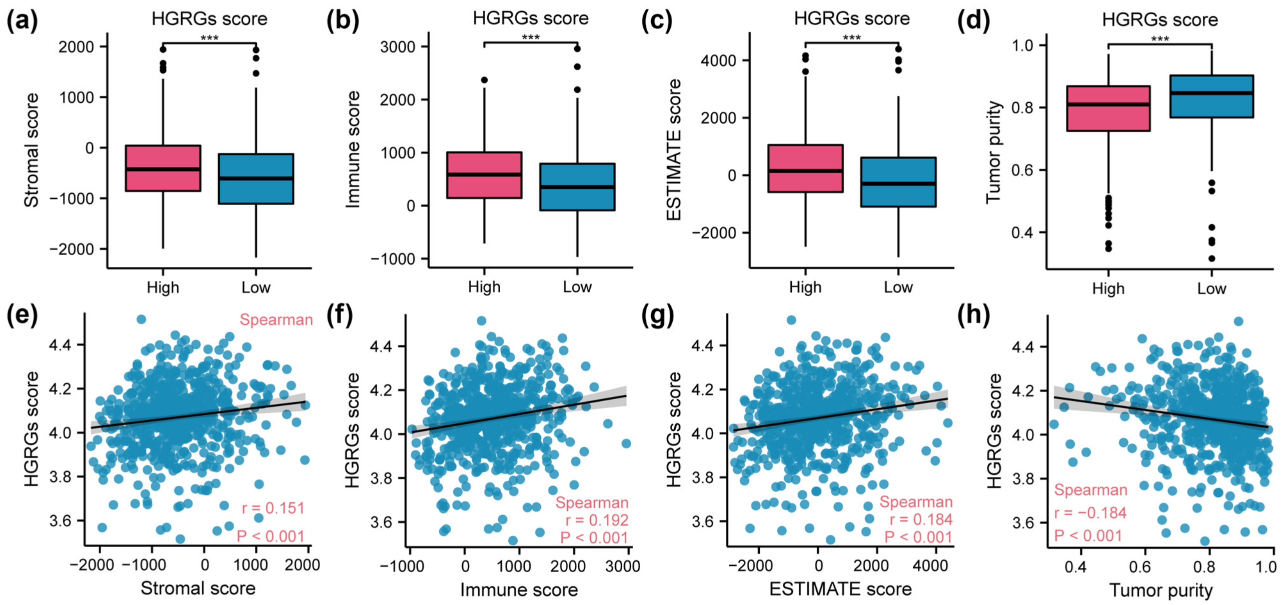

Figure S3: ESTIMATE immune evaluation between high and low HGRGs score groups in the TCGA-COADREAD dataset. (a) and (b). The comparisons of the stromal (a), immune (b), and ESTIMATE (c), scores, as well as tumor purity (d), between the two HGRGs score groups in the TCGA-COADREAD dataset. (e)–(h). The correlations of HGRGs score with stromal (e), immune (f), and ESTIMATE (g) scores, as well as tumor purity (h). ***$P < 0.001$. The absolute correlation coefficient (r) value in the scatter plots indicates the degree of correlation, where $r > 0.8$, between 0.5–0.8, between 0.3–0.5, and <0.3 indicate a strong, moderate, weak, and no correlation.

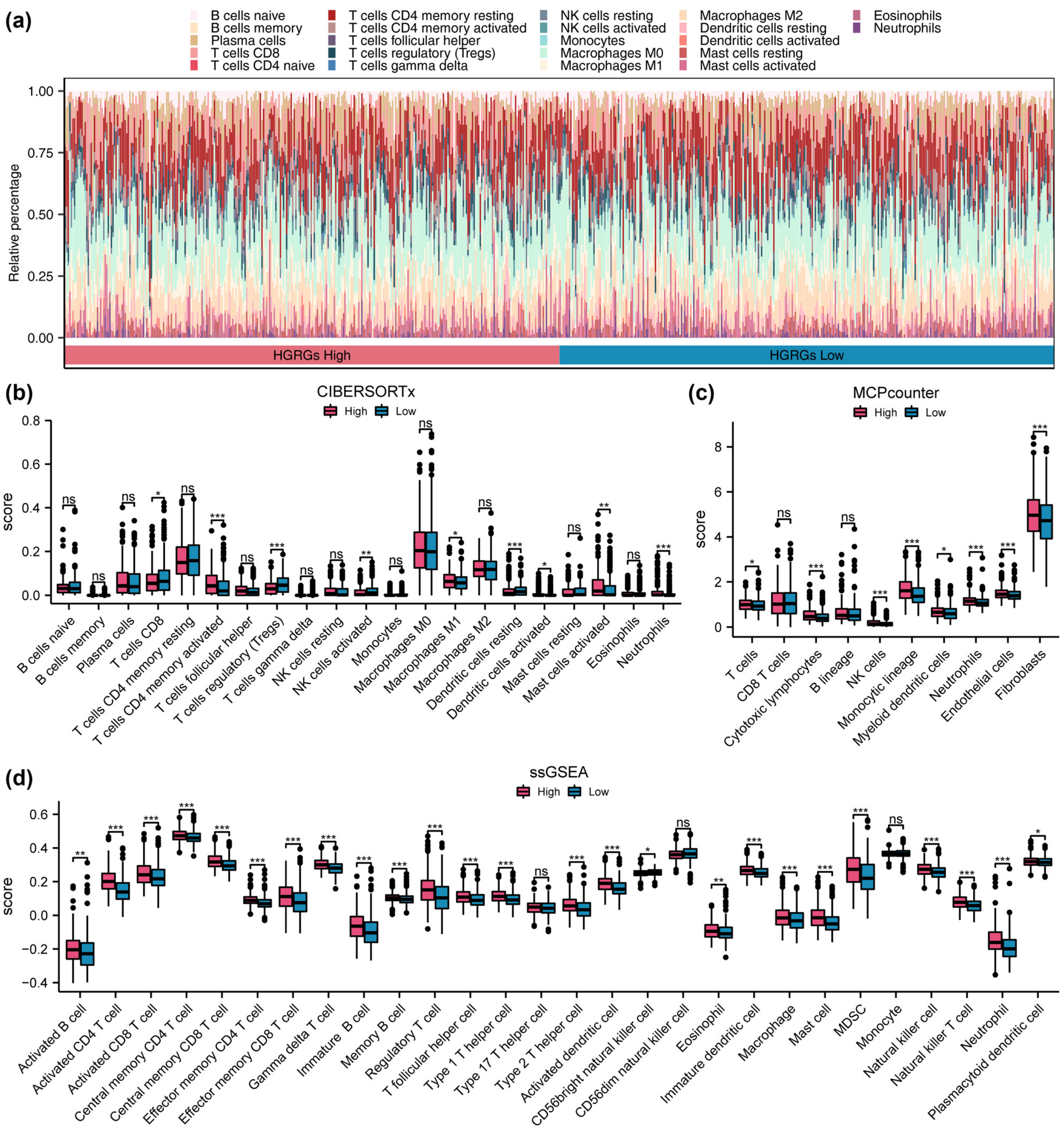

Figure S4: TCGA-COADREAD data set immune infiltration analysis stacked histogram and grouping comparison chart. (a) The relative proportions of 22 immune cell types in different cancer samples from the TCGACOADREAD dataset under the CIBERSORTx algorithm. Each color-coded bar represents a different immune cell type. (b)–(d). The comparisons of immune cell infiltration abundance between the two HGRGs score groups in the TCGA-COADREAD dataset, using CIBERSORTx (b), MCPcounter (c), and ssGSEA (d) algorithms. ns: $P \geq 0.05$, *: < 0.05, **: < 0.01, ***: $P < 0.001$.

Table S3: Signaling pathways identified with GSEA

ID	set Size	enrichment Score	NES	*p* value	*q* values
WP IL18 Signaling Pathway	272	−0.383671721	−1.519919577	0.000332336	0.003563348
WP Notch Signaling	45	−0.587457662	−1.813590616	0.000371747	0.003845655
Reactome FCERI Mediated NF KB Activation	81	0.462101368	1.622960798	0.001363327	0.010489044
Reactome Negative Regulation of Notch4 Signaling	54	0.507185454	1.664795536	0.001954397	0.013924544
KEGG Notch Signaling Pathway	47	−0.549058097	−1.706503161	0.002035153	0.014407252
Reactome TCF Dependent Signaling in Response to WNT	232	0.350841749	1.416743079	0.002733598	0.018694687
WP Focal Adhesion PI3K Akt mTOR Signaling Pathway	303	−0.336050579	−1.34523501	0.005102041	0.030590201
Reactome Cellular Response to Hypoxia	75	0.446998608	1.551140997	0.005423729	0.032119356
Reactome Signaling by WNT	329	0.314077439	1.309289252	0.005671565	0.033246015
WP Hedgehog Signaling Pathway Netpath	16	−0.705029318	−1.731603419	0.007436399	0.03999392
KEGG MAPK Signaling Pathway	267	−0.335177305	−1.325921358	0.008837752	0.045121194
PID IL4 2Pathway	61	−0.470356618	−1.532909958	0.009565857	0.04796735
Reactome Pre Notch Expression and Processing	107	0.382508459	1.403580301	0.013061412	0.059842586
KEGG JAK STAT Signaling Pathway	155	−0.369207255	−1.37569573	0.013130615	0.059865594
Reactome Glycolysis	72	0.420077892	1.449648748	0.01359483	0.061240196
Reactome MAPK6 MAPK4 Signaling	91	0.396224735	1.41907269	0.016195255	0.069143115
Reactome Repression of WNT Target Genes	14	−0.666759383	−1.588861169	0.023224044	0.088504612
Reactome Dectin 1 Mediated Noncanonical NF KB Signaling	62	0.420278898	1.415385707	0.026442308	0.097650978
WNT Signaling	89	−0.399789242	−1.387302632	0.027094922	0.099398415
Reactome Activated Notch1 Transmits Signal to the Nucleus	31	−0.52601255	−1.510878243	0.027184102	0.099514286
WP Hostpathogen Interaction of Human Corona Viruses MAPK Signaling	36	−0.497916953	−1.47312786	0.030348445	0.107593224
Reactome PI3K Events in ERBB4 Signaling	10	0.704965548	1.537945282	0.034692635	0.118263964
Reactome Hedgehog Off State	113	0.348716253	1.287142858	0.041773033	0.133757583
KEGG Hedgehog Signaling Pathway	56	−0.431542006	−1.385136492	0.042561066	0.135109401
WP PI3KAKT Signaling Pathway	339	−0.297731929	−1.203899489	0.04513606	0.140663863
Reactome Notch2 Activation and Transmission of Signal to the Nucleus	22	−0.555265099	−1.473286108	0.047262248	0.143653212
Reactome Signaling by Notch	234	0.299640642	1.210935284	0.047394541	0.143661183

Table S4: Gene symbols of 50 HGRMDEGs

Gene symbol				
**ADM**	**AK4**	**ALDOB**	**ALDOC**	**ANG**
ANGPTL4	AQP1	BNIP3	CBFA2T3	CCNA2
CD44	EDN1	ENO1	ENO2	EPAS1
FBP1	GPD1	HILPDA	HK3	HMOX1
IL1A	IRS2	JUND	KCNMB1	LDHB
MMP14	MPC1	MPC2	MYCN	NDRG1
NOS2	NR4A1	NUP210	PCK1	PDGFA
PFKP	PHGDH	PKLR	PLAT	PMAIP1
PRKACB	PTGIS	PYGB	RGCC	SLC16A1
SLC16A3	STC1	STC2	TIGAR	VEGFA

Table S5: GO and KEGG analysis results

Ontology	ID	Description	Gene Ratio	Bg Ratio	*p* value
BP	GO:0001666	response to hypoxia	19/30	286/18800	7.46276×10^{-28}
BP	GO:0071456	cellular response to hypoxia	13/30	143/18800	1.75276×10^{-20}
BP	GO:0006096	glycolytic process	5/30	81/18800	1.71568×10^{-7}
MF	GO:0016829	lyase activity	5/30	195/18410	1.4553×10^{-5}
MF	GO:0048018	receptor ligand activity	5/30	489/18410	0.001065761
MF	GO:0016830	carbon-carbon lyase activity	3/30	50/18410	7.26759×10^{-5}
KEGG	hsa04066	HIF-1 signaling pathway	7/26	109/8164	3.32479×10^{-8}
KEGG	hsa00010	Glycolysis / Gluconeogenesis	6/26	67/8164	4.92213×10^{-8}
KEGG	hsa04930	Type II diabetes mellitus	2/26	46/8164	0.009262558

Table S6: mRNAs and miRNAs of the mRNA-miRNA interaction network

mRNA	miRNA
ALDOC	hsa-miR-124-3p
ALDOC	hsa-miR-641
ALDOC	hsa-miR-874-3p
ALDOC	hsa-miR-4726-5p
CCNA2	hsa-miR-219a-5p
CCNA2	hsa-miR-410-3p
CCNA2	hsa-miR-495-3p
CCNA2	hsa-miR-454-3p
CCNA2	hsa-miR-506-5p
IL1A	hsa-miR-486-5p
IL1A	hsa-miR-545-3p
IL1A	hsa-miR-28-3p
IL1A	hsa-miR-1278
NDRG1	hsa-miR-147a
NDRG1	hsa-miR-223-3p
NDRG1	hsa-miR-490-3p
NDRG1	hsa-miR-526a
NDRG1	hsa-miR-624-3p
NDRG1	hsa-miR-628-5p
NDRG1	hsa-miR-873-5p
NDRG1	hsa-miR-1185-5p
NDRG1	hsa-miR-3150b-3p
NDRG1	hsa-miR-4640-3p
PHGDH	hsa-miR-541-3p
VEGFA	hsa-miR-10a-5p
VEGFA	hsa-miR-200b-3p
VEGFA	hsa-miR-125b-5p
VEGFA	hsa-miR-145-5p
VEGFA	hsa-miR-152-3p
VEGFA	hsa-miR-185-5p
VEGFA	hsa-miR-200c-3p
VEGFA	hsa-miR-329-3p
VEGFA	hsa-miR-509-3p
VEGFA	hsa-miR-873-5p
VEGFA	hsa-miR-3163
VEGFA	hsa-miR-3179
VEGFA	hsa-miR-4306
VEGFA	hsa-miR-2355-5p
VEGFA	hsa-miR-3924

Table S7: Univariate and multivariate Cox regression analyses

**Characteristics**	**Total (*N*)**	**Univariate analysis**		**Multivariate analysis**	
		**Hazard ratio (95% CI)**	***P* value**	**Hazard ratio (95% CI)**	***P* value**
ALDOB	643				
Low	322	Reference			
High	321	0.698 (0.492–0.991)	**0.044**	0.908 (0.609–1.354)	0.636
AQP1	643				
Low	322	Reference			
High	321	1.393 (0.982–1.975)	0.063	1.247 (0.808–1.926)	0.319
IL1A	643				
Low	322	Reference			
High	321	0.698 (0.490–0.995)	**0.047**	1.095 (0.738–1.625)	0.651
PHGDH	643				
Low	322	Reference			
High	321	1.361 (0.959–1.931)	0.085	1.626 (1.088–2.430)	**0.018**
PTGIS	643				
Low	321	Reference			
High	322	1.398 (0.986–1.981)	0.060	1.168 (0.765–1.783)	0.473
T stage	640				
T1&T2	131	Reference			
T3	435	2.047 (1.090–3.842)	**0.026**	1.798 (0.803–4.027)	0.154
T4	74	6.148 (3.045–12.415)	<**0.001**	4.920 (1.985–12.191)	<**0.001**
N stage	639				
N0	367	Reference			
N1	153	1.774 (1.131–2.781)	**0.013**	1.416 (0.823–2.436)	0.209
N2	119	3.873 (2.588–5.796)	<**0.001**	2.619 (1.523–4.505)	<**0.001**
M stage	563				
M0	474	Reference			
M1	89	3.989 (2.684–5.929)	<**0.001**	2.129 (1.318–3.437)	**0.002**
Age	643				
≤65	276	Reference			
>65	367	1.939 (1.320–2.849)	<**0.001**	2.783 (1.776–4.360)	<**0.001**
